# Comparative efficacy of non-invasive brain stimulation for post-stroke cognitive impairment: a network meta-analysis

**DOI:** 10.1007/s40520-023-02662-x

**Published:** 2024-02-12

**Authors:** Mengyu Yan, Jiarui Liu, Yiming Guo, Qingtao Hou, Jiaqi Song, Xiaoqin Wang, Weihua Yu, Yang Lü

**Affiliations:** 1 Department of Geriatrics, The First Affiliated Hospital of Chongqing Medical University, No. 1 Youyi Road, Yu Zhong District, , Chongqing, 400016 China; 2 Institute of Neuroscience, Chongqing Medical University, No. 1 Yixuayuan Road, Yu Zhong District, Chongqing, 400016 China

**Keywords:** Non-invasive brain stimulation, Stroke, Cognition, Activities of daily living, Network meta-analysis

## Abstract

**Background:**

Non-invasive brain stimulation (NIBS) is a burgeoning approach with the potential to significantly enhance cognition and functional abilities in individuals who have undergone a stroke. However, the current evidence lacks robust comparisons and rankings of various NIBS methods concerning the specific stimulation sites and parameters used. To address this knowledge gap, this systematic review and meta-analysis seek to offer conclusive evidence on the efficacy and safety of NIBS in treating post-stroke cognitive impairment.

**Methods:**

A systematic review of randomized control trials (RCT) was performed using Bayesian network meta-analysis. We searched RCT in the following databases until June 2022: Cochrane Central Register of Controlled Trials (CENTRAL), PUBMED, and EMBASE. We compared any active NIBS to control in terms of improving cognition function and activities of daily living (ADL) capacity following stroke.

**Results:**

After reviewing 1577 retrieved citations, a total of 26 RCTs were included. High-frequency (HF)-repetitive transcranial magnetic stimulation (rTMS) (mean difference 2.25 [95% credible interval 0.77, 3.66]) was identified as a recommended approach for alleviating the global severity of cognition. Dual-rTMS (27.61 [25.66, 29.57]) emerged as a favorable technique for enhancing ADL function. In terms of stimulation targets, the dorsolateral prefrontal cortex exhibited a higher ranking in relation to the global severity of cognition.

**Conclusions:**

Among various NIBS techniques, HF-rTMS stands out as the most promising intervention for enhancing cognitive function. Meanwhile, Dual-rTMS is highly recommended for improving ADL capacity.

**Supplementary Information:**

The online version contains supplementary material available at 10.1007/s40520-023-02662-x.

## Introduction

Stroke is a prominent contributor to morbidity and mortality on a global scale, imposing substantial challenges for patients, caregivers, and healthcare systems [1, 2]. As a prevalent residual complication, poststroke cognitive impairment (PSCI) is presented in nearly 70% of stroke survivors and has been implicated with unfavorable long-term outcomes [3].

Depending on the type of stroke, characteristics of the population, and time point and thresholds of assessments, the underlying cognitive profile of PSCI can be varied. Still, it is widely acknowledged that PSCI can exert deficits not only specific to stroke lesion sites but also in other cognitive networks associated with higher executional and attentional function [4], hampering the quality of life, affecting activities of daily living (ADL), and potentially elevating the risk of recurrent stroke [5]. As such, the restoration of post-stroke cognitive function has gradually received recognition equivalent to motor function [6, 7].

In the last two decades, non-invasive brain stimulation (NIBS) has emerged as a promising strategy to preserve cognitive function after stroke [8–10]. Based on the interhemispheric inhibition model, NIBS techniques collectively employ electrical or magnetic energy to achieve the balance of excitability between two hemispheres [11, 12]. The conventional NIBS techniques for PSCI include repetitive transcranial magnetic stimulation (rTMS) and transcranial direct current stimulation (tDCS).

rTMS operates by positioning a coil on the scalp to deliver brief bursts of electricity, generating a pulsating magnetic field. rTMS is categorized into high-frequency rTMS (excitatory, 3–20 Hz) and low-frequency rTMS (inhibitory, ≤ 1Hz) based on various frequency parameters [13]. Novel forms of rTMS like Theta-burst stimulation (TBS) are divided into intermittent TBS (iTBS) and continuous TBS (cTBS) [14, 15]. tDCS works by applying a weak and continuous direct current to the cerebral cortex, possessing the capability to either augment or diminish cortical excitability [16, 17]. Low-intensity direct current can alter the excitability of the cerebral cortex. Excitatory anodal tDCS and inhibitory cathodal tDCS are common neurorehabilitation modes clinically.

Although the effects of NIBS have been critically evaluated in previous meta-analyses, the incomplete findings, particularly on efficacy comparison and specific stimulation sites, have hindered the integration of NIBS into standard clinical practice [18–20]. Considering the aforementioned challenges, our objective is to conduct a thorough comparison and ranking of the effectiveness of different NIBS modalities in enhancing both post-stroke cognitive function and ADL function. Additionally, we aim to assess the domain-specific effects on various cognitive domains through the use of network meta-analysis (NMA). This approach will provide the most comprehensive and robust evidence currently available [21].

## Methods

This study followed the PRISMA guideline [22]. Furthermore, it was prospectively registered in the PROSPERO database of systematic reviews under the registration number CRD42022342903 [23].

### Search strategy

From January 2012 to June 2022, a systematic search was performed in three electronic databases, including PubMed, Embase, and the Cochrane Library. There were no language or other restrictions. The comprehensive strategy, including search terms tailored for each database, is available in Supplementary Appendix 2.

### Inclusion and exclusion criteria

Two independent investigators (MY and JL) carried out the selection of records based on screening criteria that involved filtering through titles, abstracts, and full texts. If any discrepancies arose, a third reviewer (YL) was consulted for resolution.

The inclusion criteria for the studies were as follows: (1) participants: individuals diagnosed with a stroke, confirmed through standardized scales and neuroimaging; (2) intervention: Non-invasive brain stimulation (NIBS) modes, including rTMS, tDCS, and other variants; (3) comparison: placebo therapy or sham stimulation. In cases where combined interventions were utilized, the control group received the same non-invasive brain stimulation component of the intervention (e.g., brain stimulation plus conventional rehabilitation therapy vs. sham plus conventional rehabilitation therapy); (4) outcome: Changes in scale values or performance on cognitive and ADL tasks after therapy, including Mini-Mental State Examination (MMSE), Montreal Cognitive Assessment (MoCA), Rivermead Behavioural Memory Test (RBMT), Trail Making Test (TMT), Line Bisection Test (LBT), Star Cancellation Test(SCT), Catherine Bergego Scale(CBS), Modified Barthel Index (MBI), Barthel Index (BI), Functional Independence Measure (FIM) and National Institutes of Health Stroke Scale (NIHSS) [24–31], with studies reporting sufficient information to compute common effect size statistics (i.e., mean and standard deviations [SD], exact *F*-, *p*-, *t*-, or *z*-values); (5) study design: Randomized controlled trials (RCTs) involving adult participants (≥ 18 years). Studies were not considered for inclusion if they met any of the following exclusion criteria: (1) publication in the form of abstracts; (2) implementation of interventions that were irrelevant or imbalanced across different groups (e.g., invasive interventions); (3) failure to report pre-post changes in cognitive performance or inability to calculate these changes based on the available data (e.g., inconsistent results not following the consolidated standards using computerized measurement systems); and (4) study types that could not provide reliable data according to GRADE (Grading of Recommendations, Assessment, Development, and Evaluation) methodology [32], the initial quality assessment corresponds to the study design, i.e., “high” for experimental studies (eg, randomized clinical trials [RCTs]) and “low” for observational studies (e.g., case reports and cohort studies), including case reports and cohort studies.

### Data extraction

Two reviewers (MY and JL) extracted data from eligible studies independently and assessed by another experienced investigator (YL). A pre-specified form showed demographic features, study characteristics, clinical information and stimulation parameters. The outcome data of cognition level and activities of daily living function were collected from the main text, tables and supplementary materials. The results of outcome assessment values in the figures were carried out using Engauge Digitizer (version 12.1).

### Quality assessment

Two independent reviewers (JS and XW) assessed the quality of the included studies using the Cochrane collaboration’s tool for assessing the risk of bias in randomized trials (RoB) in Review Manager (version 5.4.1). RoB was based on the Cochrane Handbook recommendations and explored sources of bias based on seven dimensions, namely the risk of bias in six domains: selection bias, performance bias, detection bias, attrition bias, reporting bias and other bias [33]. (as shown in Supplementary Figs. 3 and 4).

### Primary and secondary outcomes

Pre-post changes in the global severity of cognitive impairment were considered as the primary outcomes. The primary outcomes of global cognition severity were quantified using MMSE and MoCA [24]. The assessment of subdomain scales, such as executive function, memory, and perception, served as secondary outcomes in the study. These scales included RBMT [25], TMT [26], LBT, SCT, CBS [27] and Motor-Free Visual Perception Test (MVPT).

Secondary outcomes in this study also included the MBI [28], BI [29], FIM [30], and NIHSS) [31]. These scales were used to assess the overall severity of daily living abilities and stroke. Further details about these scales can be found in Supplementary Appendix 3. Adverse events and dropouts are also summarized in Supplementary Appendix 4.

### Statistical analysis

An NMA based on a Bayesian random-effects framework was performed using R (version 4.1.3, gemtc package (1.0–1)). In this study, a Markov chain Monte Carlo method was employed to combine the direct and indirect comparisons of interventions, with the number of chains set at four. Gibbs sampling [34] was according to 20,000 iterations by removing 5,000 iterations in the burn-in phase. The effect size of therapeutic efficacy for continuous variables, as measured by scales, was evaluated using the pooled mean differences (MDs) of pre-post cognitive changes along with their corresponding 95% credible intervals (CrIs). Given that some studies did not directly report change values, we derived the standard deviations (SDs) of pre-post changes by utilizing the original datasets acquired from online supplements or obtained through requests made to the respective authors. In cases where the aforementioned methodologies were not available, we employed a simple imputation approach to estimate the SD values, as outlined in the Cochrane Handbook for Systematic Reviews of Interventions [35]. To rank the therapy efficacies of each outcome, the surface under the cumulative ranking curve (SUCRA) was computed, with a higher SUCRA value, approaching 100%, suggesting a higher likelihood of an intervention ranking among the top positions [36]. The level of statistical significance was set to *p* < 0.05.

### Inconsistency of model

To assess inconsistency, the 'mtc.nodesplit' method was employed, and a significance level of *p* < 0.05 was utilized to determine whether the inconsistency was statistically significant.

## Results

### Study selection

**Fig. 1:**
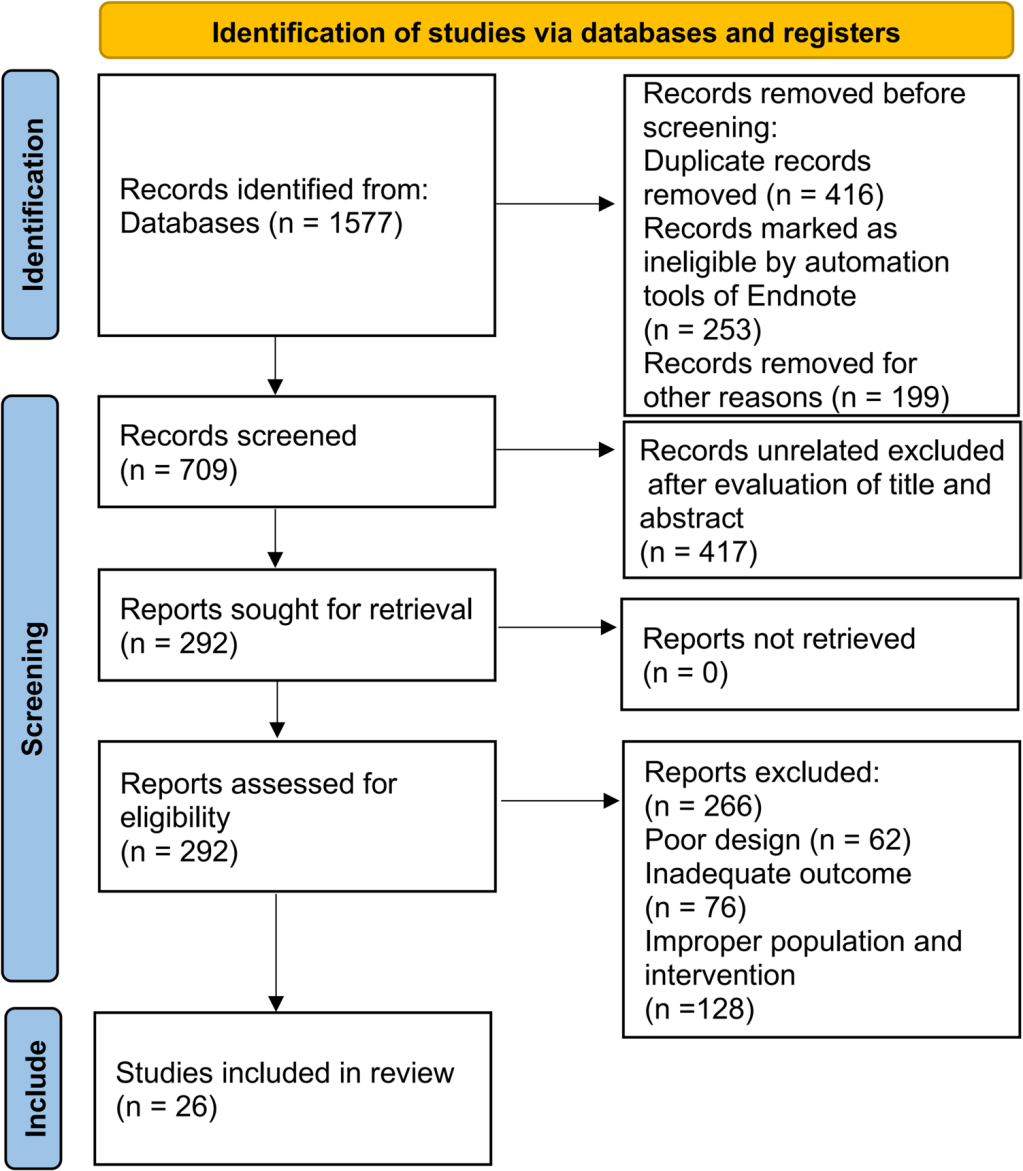
Flow diagram illustrating the selection of studies for inclusion in the network meta-analysis

The study selection process is presented in Fig. 1. A total of 1577 records were initially obtained from databases. After removing 416 duplicate records, 1161 records remained. Following the screening of titles and abstracts to exclude irrelevant data, 292 full-text reports were selected for further assessment of eligibility. Trials conducted by a specific research team were included as long as they involved different populations or interventions within the study cohort. A total of 26 RCT reports were included in the meta-analysis.

### Study characteristics

The detailed characteristics of individual studies are listed in Table 1. This meta-analysis included 1062 patients with PSCI. The average proportion of females in the included studies was 42.5%, and the average age of the participants was 58.91 ± 12.14 years. Among the enrolled patients, a small subset (25%, 5 out of 20) were in the chronic phase of stroke recovery (more than 6 months), while 70% (14 out of 20) were in the subacute phase (7 days to 6 months). By the way, only one trial [37] was in the acute phase and one trial [48] was categorized as both subacute and chronic post-stroke. Five trials [42, 46, 53, 56, 57] did not provide information about the duration of the disease course. Two clinical trials [38, 53] did not report participant ages.

**Table 1:** Demographic and clinical characteristics of the included studies

Study (year)	Study design	Group	*n*	Age	%*F*	Duration of illness
rTMS						
Qingmei Chen et al. (2021) [37]	Double-Blind	Dual-rTMS-M1	25	55.85 ± 16.51	28	7.36 ± 3.93 (d)
		HF-rTMS-M1	25	59.14 ± 14.15	28	6.07 ± 3.93 (d)
		LF-rTMS-M1	25	57.28 ± 18.87	32	7.72 ± 5.50 (d)
		Placebo	25	63.21 ± 16.51	28	6.25 ± 4.32 (d)
Hong Li et al. (2021) [10]	Single-Blind	LF-rTMS-DLPFC	33	61.79 ± 5.51	36	28.64 ± 12.60 (d)
		Placebo	32	59.47 ± 6.75	41	27.78 ± 11.01 (d)
Fangzhou Yu et al. (2021) [38]	NM	HF-rTMS-DLPFC	60	55.91 ± 8.76	NM	27.58 ± 4.51 (d)
		Placebo	55	55.75 ± 9.02	NM	27.02 ± 5.20 (d)
Mingyu Yin et al. (2020) [39]	Double-Blind	HF-rTMS-DLPFC	16	56.69 ± 12.92	12.5	63.95 ± 49.18 (d)
		Placebo	18	58.17 ± 11.27	11.1	63.84 ± 44.25 (d)
Yamei Li et al. (2020) [40]	Double-Blind	HF-rTMS-DLPFC	15	65.47 ± 3.68	53.3	22.73 ± 8.05 (d)
		Placebo	15	64.53 ± 4.72	40	19.13 ± 7.95 (d)
Yuanwen Liu et al. (2020) [41]	Double-Blind	HF-rTMS-DLPFC	29	58.55 ± 6.24	65.5	8.79 ± 1.84 (m)
		Placebo	29	57.69 ± 7.25	44.8	8.62 ± 1.84 (m)
Shole Vatanparasti et al. (2019) [42]	Single-Blind	cTBS-PPC	7	65.5 ± 10.2	30	NM
		Placebo	7	67.5 ± 8.4	30	NM
Thomas Nyffeler et al. (2019) [43]	Double-Blind	Placebo	10	70.60 ± 11.44	30	25.8 ± 11.26 (d)
		cTBS-PPC	10	67.80 ± 10.13	50	26.8 ± 20.89 (d)
		cTBS-PPC	10	74.30 ± 10.23	40	22.90 ± 10.34 (d)
Sang Beom Kim et al. (2018) [44]	NM	Placebo	10	66.6 ± 12.2	50	24.5 ± 22.4 (d)
		LF-rTMS-PPC	10	62.5 ± 16.5	50	15.3 ± 9.8 (d)
Ayhan Askin et al. (2017) [45]	Single-Blind	LF-rTMS-M1	20	58.80 ± 12.02	75	24.35 ± 15.39 (m)
		Placebo	20	56.75 ± 11.46	70	28.35 ± 15.34 (m)
Ko Un Kim et al. (2017) [46]	NM	LF-rTMS-PPC	22	52.6 ± 10.6	18.2	NM
		Placebo	22	64.3 ± 11.5	40.9	NM
Koichi Hosomi et al. (2016) [47]	Double-Blind	HF-rTMS-M1	18	62.4 ± 15.5	44	46.1 ± 8.7 (d)
		Placebo	21	63.2 ± 12.5	38	45.1 ± 9.5 (d)
Haitao Lu et al. (2015) [48]	Double-Blind	LF-rTMS-DLPFC	19	42.5 ± 12.3	36.8	161.03 ± 268.32 (d)
		Placebo	21	47.3 ± 11.8	38.1	132.89 ± 211.48 (d)
Wei Yang et al. (2015) [49]	NM	LF-rTMS-PPC	9	46.72 ± 13.11	33.3	100.96 ± 38.52 (d)
		HF-rTMS-PPC	10	48.01 ± 12.25	60	107.52 ± 39.24 (d)
		cTBS-PPC	9	49.45 ± 10.78	55.6	104.85 ± 36.38 (d)
		Placebo	10	47.70 ± 11.81	70	105.91 ± 37.59 (d)
Hyun Gyu Cha et al. (2015) [50]	Double-Blind	LF-rTMS-PPC	10	59.8 ± 9.9	50	4.4 ± 0.2 (w)
		Placebo	10	56.7 ± 8.2	40	4.9 ± 0.3 (w)
Bo Ryun Kim et al. (2013) [51]	Double-Blind	LF-rTMS-PPC	9	68.6 ± 14.4	44.4	14.2 ± 4.7 (d)
		HF-rTMS-PPC	9	64.1 ± 10.3	55.6	14.3 ± 3.6 (d)
		Placebo	9	68.3 ± 6.5	33.3	16.4 ± 8.5 (d)
Dario Cazzoli et al. (2012) [52]	Double-Blind	cTBS-PPC	8	58 ± 11.02	29.2	26.63 ± 21.75 (d)
		cTBS-PPC	8			
		Placebo	8			
G. Koch et al. (2012) [53]	Double-Blind	cTBS-PPC	9	NM	NM	NM
		Placebo	9	NM	NM	NM
tDCS						
Danielle De S. Boasquevisque et al. (2021) [54]	Double-Blind	c-tDCS-M1	15	61.8 ± 15	53.3	35.2 ± 18 (d)
		Placebo	15	61.9 ± 17.9	26.7	28.4 ± 13.5 (d)
Hussien Ahmed Shaker et al. (2018) [55]	Single-Blind	Dual-tDCS-DLPFC	20	54.45 ± 4.68	0	14.05 ± 1.53 (m)
		Placebo	20	53.05 ± 6.32	0	16.55 ± 2.78 (m)
Hosseinzadeh et al. (2018) [56]	Double-Blind	Placebo	25	59 ± 8	52	NM
		Sham	25	59 ± 7	52	NM
		a-tDCS-STG	25	58 ± 8	48	NM
		c-tDCS-STG	25	60 ± 7	52	NM
You Gyoung Yi et al. (2016) [57]	NM	a-tDCS-M1	10	63.0 ± 8.5	70	NM
		Dual-tDCS-PPC	10	61.6 ± 12.2	80	NM
		placebo	10	61.7 ± 9.5	60	NM
Ko Un Kim et al. (2016) [58]	NM	a-tDCS-PPC	15	58.7 ± 12.6	66.7	14.6 ± 6.0 (m)
		Placebo	15	51.9 ± 10.7	60	14.5 ± 6.9 (m)
Gi Jeong Yun et al. (2015) [59]	Double-Blind	a-tDCS-FTP-L	15	60.9 ± 12.9	60	42.2 ± 31.9 (d)
		a-tDCS-FTP-R	15	58.9 ± 15.0	53.3	38.1 ± 27.0 (d)
		Placebo	15	68.5 ± 14.6	53.3	39.5 ± 29.6 (d)
See Hyun Park et al. (2013) [8]	Double-Blind	Dual-tDCS-DLPFC	6	65.3 ± 14.3	66.7	29.0 ± 18.7 (d)
		Placebo	5	66.0 ± 10.8	40	25.2 ± 17.5 (d)
Hyuk Sunwoo et al. (2013) [60]	NM	Dual-tDCS-PPC	10	62.6 ± 13.3	40	27.8 ± 60.4 (m)
		a-tDCS-PPC				
		Placebo				

*NM* not mentioned, *d* day, *w* week, *m* month, *year* year of publication, *design* study design, *group* stimulation type-stimulation site, *n* number of participants per group, *Age* mean ± standard deviation if available, *%F* proportion of females, *Duration of illness* mean ± standard deviation if available, *HF-* high frequency, *LF-* low frequency, *cTBS* continuous theta burst stimulation, *a-* anodal, *c-* cathodal, *DLPFC* dorsolateral prefrontal cortex, *STG* superior temporal gyrus, *FTP* fronto-temporal region, *PPC* posterior parietal cortex, *M1* primary motor cortex, *rTMS* repetitive transcranial magnetic stimulation, *tDCS* transcranial direct current stimulation

Concerning the details of the therapy interventions, each therapy lasted a mean of 15.8 ± 5.7 (6–30) sessions, and rTMS demonstrated to be the most frequent form of NIBS for addressing cognitive and functional deficits following stroke. Out of the total number of trials, 18 (69.2%) focused on rTMS, encompassing 13 conventional rTMS trials, 4 TBS trials, and 1 trial [49] combining both types of stimulation. A significant proportion of the trials focused on measuring the primary outcomes related to the global severity of post-stroke cognitive impairments. Various cognition scales, such as MMSE, MoCA, TMT, RBMT, MVPT, LBT, SCT, and CBS, were employed for outcome assessments. Additionally, outcome measurements also included global severity assessments of ADL and stroke using scales such as BI, MBI, FIM, and NIHSS. Detailed information can be found in Supplementary Table 1.

### Risk of bias

The risk of bias in the included studies is summarized in Supplementary Fig. 3. Across the seven domains of the PRISMA RoB tool, two domains were identified to have a high risk of bias (as shown in Supplementary Fig. 4).

### Network geometry of intervention

**Fig. 2:**
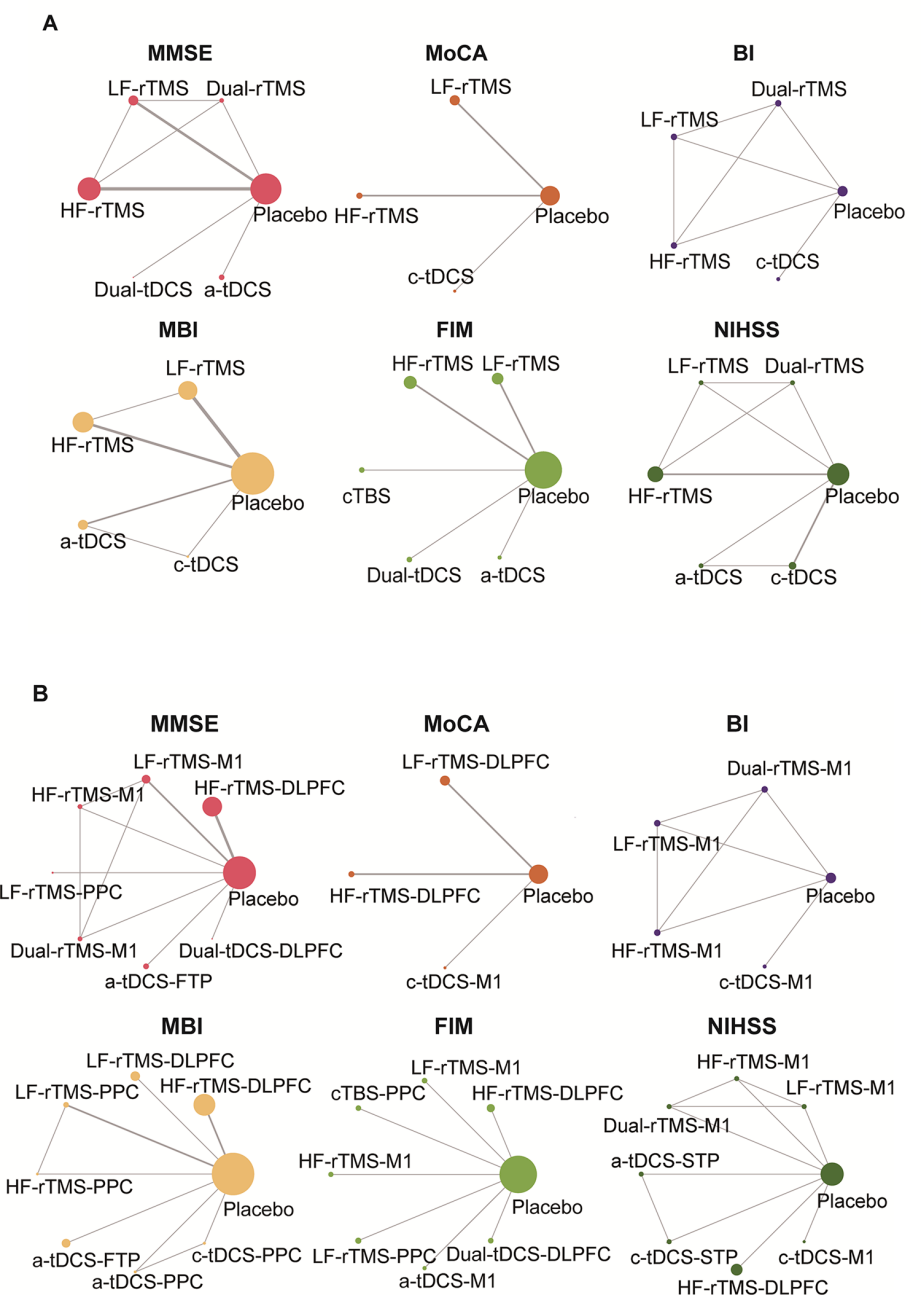
Network geometry of interventions across cognition and ADL function in the short-term assessment**.** **A** Gross network plots of NIBS modalities for global severity of cognition and ADL function as defined by stimulation parameters; **B** Refined network plots of NIBS subtypes for global severity of cognition and ADL function by targeted stimulation location. Each node in the diagram represents an intervention, with its size reflecting the sample size of patients involved. The edges connecting the nodes represent direct comparisons between interventions, and their width is proportional to the number of trials conducted for each specific comparison. H*F*- high frequency, *LF-* low frequency, *cTBS* continuous theta burst stimulation, *a-* anodal, *c-* cathodal, *DLPFC* dorsolateral prefrontal cortex, *STG* superior temporal gyrus, *FTP* fronto-temporal region, *PPC* posterior parietal cortex, *M1* primary motor cortex, *NIBS* non-invasive brain stimulation, *ADL* activities of daily living, *rTMS* repetitive transcranial magnetic stimulation, *tDCS* transcranial direct current stimulation

Figure 2 presents the geometry of the treatment networks across each cognition domain in the short-term assessment of the primary and secondary outcomes. As shown in Fig. 2A, the trials reporting cognition function measured by MMSE contained six interventions and eight pairs of direct comparisons. Another scale called MoCA contained four interventions and three edges. Furthermore, Fig. 2A illustrates the network geometry of ADL, which consists of five nodes and seven edges for BI, five nodes and six edges for MBI, and six nodes and five edges for FIM. The global severity of the stroke, as measured by NIHSS, is represented by a network with six nodes and nine edges. Across the subdomains (see Supplementary Fig. 1A), the visual perception network showed by LBT and SCT had the highest number of nodes (seven interventions) and edges (eleven comparisons). Other subdomain network geometry included TMT (four nodes, four edges) for executive function, RBMT (three nodes, two edges) for memory function, and MVPT (five nodes, six edges) and CBS for perception function (six nodes, seven edges).

Refined networks subdivided by targeted brain regions are presented in Fig. 2B and Supplementary Fig. 1B.

### Efficacy across cognition, living function and stimulation regions according to pooled MDs

**Fig. 3:**
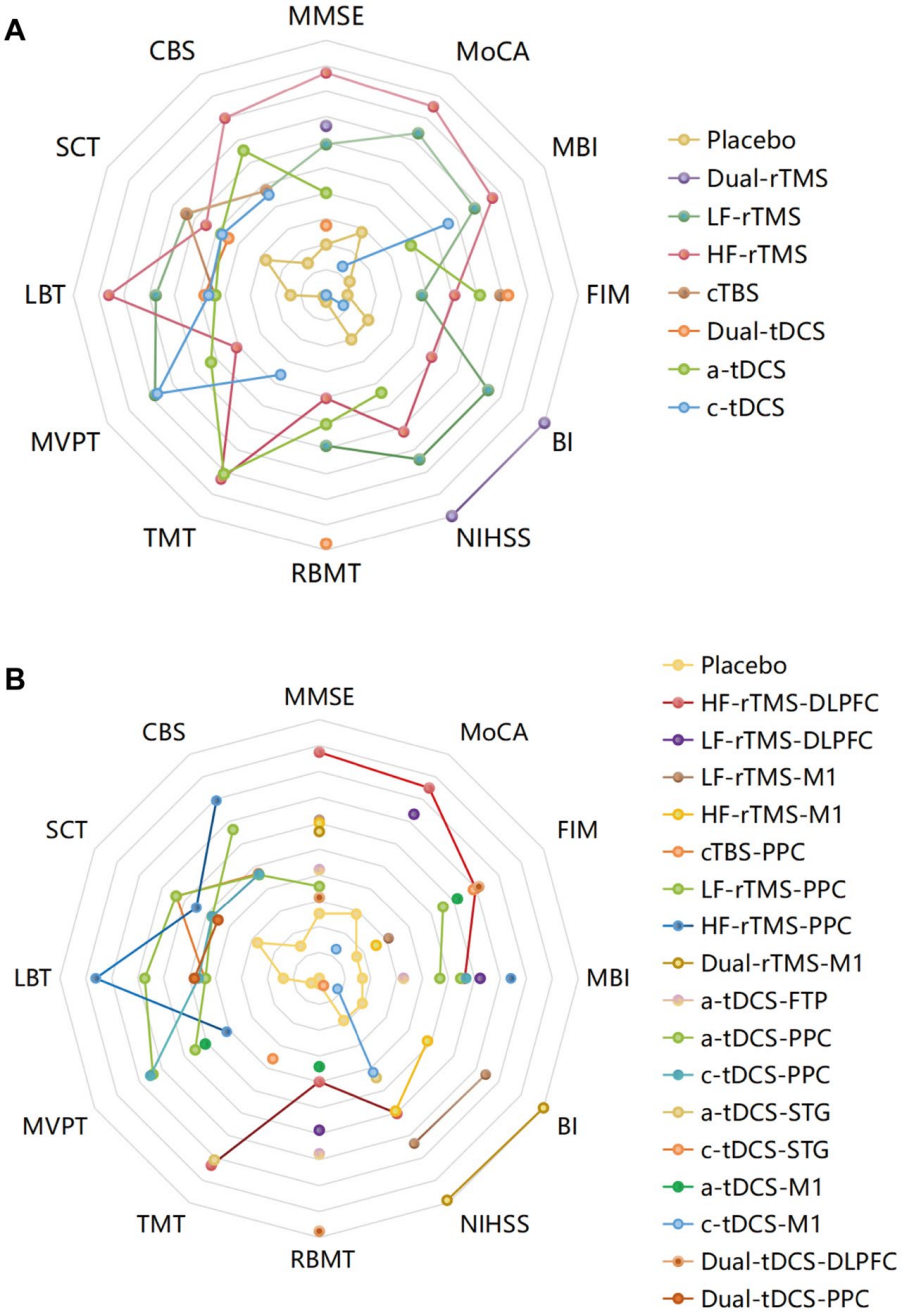
Rankings of the effects of different NIBS modalities on cognition recovery measured with SUCRAs (surface under the cumulative ranking curve). SUCRAs (values range from 0 to 1) for each cognition domain and ADL function are shown in radar graphs as defined by stimulation parameters (**A**) and by targeted stimulation location (**B**). The SUCRA value represents the likelihood of an intervention being ranked as the highest. Colored lines indicate the effect sizes of interventions compared to placebo. *HF-* high frequency, *LF-* low frequency, *cTBS* continuous theta burst stimulation, *a-* anodal, *c-* cathodal, *DLPFC* dorsolateral prefrontal cortex, *STG* superior temporal gyrus, *FTP* fronto-temporal region, *PPC* posterior parietal cortex, *M1* primary motor cortex, *NIBS* non-invasive brain stimulation, *ADL* activities of daily living, *rTMS* repetitive transcranial magnetic stimulation, *MD* mean difference, *tDCS* transcranial direct current stimulation

Regarding the measurement of cognition and living function, the present review included two interventions (rTMS and tDCS). There were 26 studies included in our network meta-analysis enrolling 1062 stroke patients. Rankings of the effects of NIBS modalities measured with SUCRAs are shown in Fig. 3.

### The global severity of cognition

**Fig. 4:**
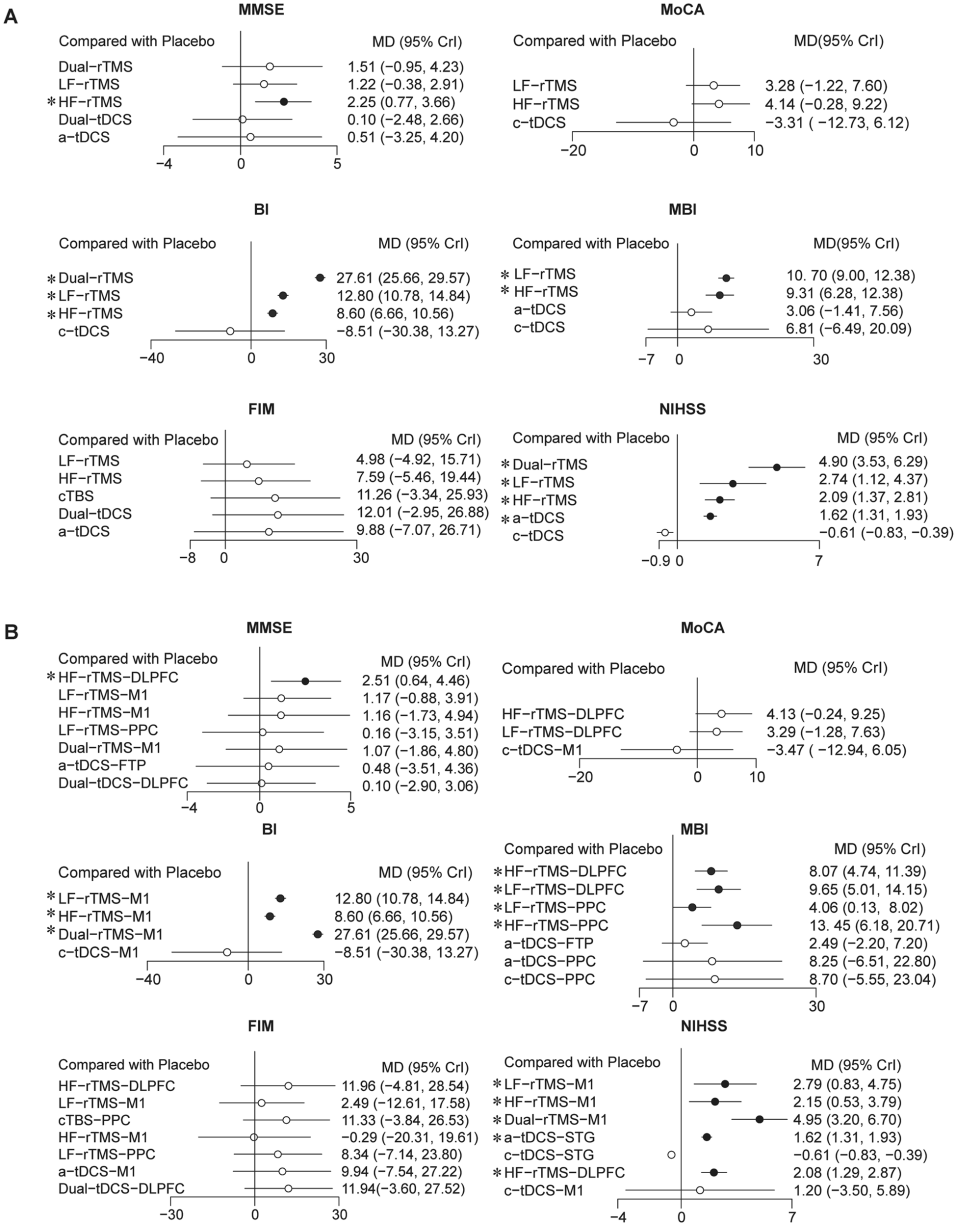
Forest plots of network meta-analyses compared with placebo across various cognition domains and ADL function, pooling the effects of NIBS modalities (number of trials ≥ 2). **A** Forest plots of NIBS modalities for global severity of cognition and ADL function as defined by stimulation parameters; **B** forest plots of refined NIBS subtypes for global severity of cognition and ADL function by targeted stimulation location. *HF-* high frequency, *LF-* low frequency, *cTBS* continuous theta burst stimulation, *a-* anodal, *c-* cathodal, *DLPFC* dorsolateral prefrontal cortex, *STG* superior temporal gyrus, *FTP* fronto-temporal region, *M1* primary motor cortex, *NIBS* non-invasive brain stimulation, *ADL* activities of daily living, *rTMS* repetitive transcranial magnetic stimulation, *MD* mean difference, *tDCS* transcranial direct current stimulation

Combining the forest plots and the SUCRA value, it can be suggested that HF-rTMS (MD 2.25 [95% CrI 0.77, 3.66]) has shown to be the most effective therapy in improving global cognitive severity. None of the interventions demonstrated significant efficacy in improving the outcome of MoCA. HF-rTMS (with a mean difference of 4.14 [− 0.28, 9.22]) was ranked among the other treatment modalities (refer to Fig. 4A and Table 2).

**Table 2:** Rankings of different interventions

Assessments	Rankings of different interventions
MMSE	HF-rTMS[2.25(0.77,3.66)] > Dual-rTMS > LF-rTMS > a-tDCS > Dual-rTMS > Placebo
	HF-rTMS-DLPFC[2.51(0.64,4.46)] > LF-rTMS-M1 > HF-rTMS-M1 > Dual-rTMS-M1 > a-tDCS-FTP > LF-rTMS-PPC > Dual-tDCS-DLPFC > Placebo
MoCA	HF-rTMS > LF-rTMS > Placebo > c-tDCS
	HF-rTMS-DLPFC > LF-rTMS-DLPFC > Placebo > c-tDCS-M1
BI	Dual-rTMS[27.61(25.66,29.57)] > LF-rTMS[12.80(10.78,14.84)] > HF-rTMS[8.60(6.66,10.56)] > Placebo > c-tDCS
	Dual-rTMS-M1[27.61(25.66,29.57)] > LF-rTMS-M1[12.80(10.78,14.84)] > HF-rTMS-M1[8.60(6.66,10.56)] > Placebo > c-tDCS-M1
MBI	LF-rTMS[10.70(9.00,12.38)] > HF-rTMS[9.31(6.28,12.38)] > c-tDCS > a-tDCS > Placebo
	HF-rTMS-PPC[13.45(6.18,20.71)] > HF-rTMS-DLPFC[9.65(5.01,14.15)] > HF-rTMS-DLPFC[8.07(4.74,11.39)] > LF-rTMS-PPC[4.06(0.13,8.02)] > c-tDCS-PPC > a-tDCS-PPC > a-tDCS-FTP > Placebo
FIM	Dual-tDCS > cTBS > a-tDCS > HF-rTMS > LF-rTMS > Placebo
	HF-rTMS-DLPFC > Dual-tDCS-DLPFC > cTBS-PPC > a-tDCS-M1 > LF-rTMS-PPC > LF-rTMS-M1 > Placebo > HF-rTMS-M1
NIHSS	Dual-rTMS[4.90(3.53,6.29)] > LF-rTMS[2.74(1.12,4.37)] > HF-rTMS[2.09(1.37,2.81)] > a-tDCS[1.62(1.31,1.93)] > Placebo > c-tDCS
	Dual-rTMS-M1[4.95(3.20,6.70)] > LF-rTMS-M1[2.79(0.83,4.75)] > HF-rTMS-M1[2.15(0.53,3.79)] > a-tDCS-STG[1.62(1.31,1.93)] > HF-rTMS-DLPFC[2.08(1.29,2.87)] > c-tDCS-M1 > Placebo > c-tDCS-STG

*HF-* high frequency, *LF-* low frequency, *cTBS* continuous theta burst stimulation, *a-* anodal, *c-* cathodal, *DLPFC* dorsolateral prefrontal cortex, *STG* superior temporal gyrus, *FTP* fronto-temporal region, *PPC* posterior parietal cortex, *M1* primary motor cortex, *rTMS* repetitive transcranial magnetic stimulation, *tDCS* transcranial direct current stimulation

Regarding targeted brain regions, it can be considered that HF-rTMS-DLPFC (2.51 [0.64, 4.46]) is the best among these interventions in improving the global severity of cognition from MMSE. Again, none of the interventions also show apparent efficacy in enhancing global cognitive function. However, like the results from MMSE, based on the SUCRA value, HF-rTMS-DLPFC (4.13 [− 0.24, 9.25]) was also ranked among these modes in the measurement of MoCA (see Fig. 4B and Supplementary Table 2).

### ADL function

Considering the BI index, Dual-rTMS had a superior effect size (27.61 [25.66, 29.57]), and significant advantages were also observed with LF-rTMS (12.80 [10.78, 14.84]) and HF-rTMS (8.60 [6.66, 10.56]). Same results come from the MBI index, HF-rTMS (9.31 [6.28, 12.38]) and LF-rTMS (10.70 [9.00, 12.38]) had priority among these modes. LF-rTMS (2.74 [1.12, 4.37]), HF-rTMS (2.09 [1.37, 2.81]), and a-tDCS (1.62 [1.31, 1.93]) had the same advantages on the stroke recovery of the NIHSS scale (refer to Fig. 4A and Table 2).

In terms of the targeted brain regions, among the specific modes of NIBS, Dual-rTMS-M1 exhibited the most favorable effect size (27.61 [25.66, 29.57]) in improving BI. HF-rTMS-DLPFC (8.07 [4.74, 11.39]), LF-rTMS-DLPFC (9.65 [5.01, 14.15]), LF-rTMS-PPC (mean difference 4.06 [95% CrI 0.13, 8.02]), and HF-rTMS-PPC (13.45 [6.18, 20.71]) all exhibited advantages in improving MBI. Furthermore, Dual-rTMS-M1 (4.95 [3.20, 6.70]), LF-rTMS-M1 (2.79 [0.83, 4.75]), HF-rTMS-M1 (2.15 [0.53, 3.79]), HF-rTMS-DLPFC (2.08 [1.29, 2.87]), and a-tDCS-STG (1.62 [1.31, 1.93]) all showed improvements in the rehabilitation process of stroke according to NIHSS (see Fig. 4B and Supplementary Table 2).

### Subdomains of cognition

LF-rTMS (3.89 [2.05, 5.95]) had impressive privileges in the MVPT scale. HF-rTMS (19.12 [3.52, 34.94]), LF-rTMS (13.03 [1.30, 24.78]) had considerable improvement among these NIBS modes in the results of the LBT scale. Three modes, including HF-rTMS (1.85 [0.94, 2.76]), a-tDCS (1.77 [1.54, 2.00]), and c-tDCS (1.17 [0.92, 1.42]), had the same positive influence in the executive function of TMT scale. Only LF-rTMS (2.81 [1.47, 4.14]) offered benefits in the results of the RBMT scale (see Supplementary Fig. 2A).

Regarding targeted brain regions, LF-rTMS-PPC (3.92 [1.83, 6.21]) had a moderate privilege in the MVPT scale. HF-rTMS-PPC (19.12 [3.34, 35.24]), LF-rTMS-PPC (13.08 [1.20, 25.18]) had considerable improvement among these NIBS modes in the results of the LBT scale. Three modes, including HF-rTMS- DLPFC (1.86 [0.94, 2.77]), a-tDCS-STG (1.77 [1.53, 2.00]), and c-tDCS-STG (1.17 [0.92, 1.42]), had the same positive influence in the executive function of TMT scale. In addition, LF-rTMS-DLPFC (2.81 [1.48, 4.14]) offered evident benefits in the results of the RBMT scale (see Supplementary Fig. 2B).

### Adverse events and dropouts

Participants in both rTMS and tDCS trials reported experiencing pricking sensations and tingling. Furthermore, a small percentage of trials (11%, 3 out of 26) [37, 40, 48] documented the occurrence of mild symptoms, such as temporary headaches and dizziness in the rTMS sessions. No severe adverse events were documented in any of the trials included in the analysis. Overall, both rTMS and tDCS are considered relatively safe, as no patients withdrew from the trials due to serious adverse effects.

### Evaluation of the inconsistency

Test results for outcomes that met the test criteria were shown by stimulation type and by targeted stimulation location (See Supplementary Fig. 5).

## Discussion

The present NMA assessed the efficacy of seven NIBS modalities across 1062 patients with cognition deficits at various levels. Most NIBS research on these symptoms finds positive effects across several cognitive subdomains. HF-rTMS ranks the highest in improving the global severity of cognition. Considering the subdomains, LF-rTMS is ranked higher in improving memory and unilateral spatial neglect (USN). a-tDCS has a positive effect on the global severity of stroke, USN and executive function. Taken together, Dual-rTMS is recommended for enhancing ADL function. Refined NMA based on locations find that DLPFC stimulation has a clear advantage, especially regarding global severity. Also, PPC is the best choice for USN. Moreover, M1 stimulation exhibits a significant influence on the BI and NIHSS scales. Additionally, the STG site plays a vital role in executive function.

### Domain-specific rankings across cognition aspects and ADL

We focus on examining the effectiveness of various treatments throughout the stroke recovery process. The enhancement of cognition and ADL capacity could potentially have a broad positive impact on stroke patients [41]. Therefore, we rank the efficacy of these functions in terms of stroke.

We find that the overall effects tend to be more favorable for bilateral stimulation, particularly in relation to ADL function. According to a study by Qingmei Chen et al. [37], the application of HF-rTMS and LF-rTMS on both the affected and unaffected hemispheres provides additional therapeutic benefits for functional recovery. Other studies have shown alignment, where Dual-rTMS exerts significant protective effects on the risk of worsening cognitive decline [61]. We speculate that applying Dual-rTMS can alleviate the interhemispheric inhibition interaction and promote recruiting collaborative potential between two hemispheres motor cortices across varying injury extents when comparing bilateral stimulation to unilateral stimulation. Regardless, more studies are required to explore the unidentified neurophysiological mechanisms of synergistic advantages in Dual-rTMS.

The NMA suggests a favorable effect of a-tDCS in the global severity of a stroke, USN and executive function. The advantageous mechanism of anodal transcranial direct current stimulation (a-tDCS), similar to HF-rTMS mentioned previously. In the NMA of Bernhard Elsner et al. [62], c-tDCS is the most promising treatment option to improve ADL capacity and arm function in patients with stroke, which is contradictory to our results partly. However, this NMA included a large proportion of c-tDCS studies, which may affect the conclusion of the superiority of c-tDCS with the advantages of publication bias. In general, there is controversy surrounding the effectiveness of different types of tDCS in stroke recovery.

Concerning stimulation modes, rTMS works at more localized areas under the coil, whereas tDCS stimulates less focal and more broadly brain regions. Meanwhile, tDCS presents a more cost-effective and convenient option compared to rTMS, making it a viable alternative for clinical intervention.

### Selection of targeted brain regions contributes to rehabilitation

There is insufficient evidence to precisely address the underlying mechanism of rTMS and tDCS in neurorehabilitation among PSCI patients. Various factors, such as distinct pathophysiological mechanisms, heterogeneity, may influence the outcomes of stimulation types. Therefore, brain region selection is crucial for increasing functional connectivity within the brain network.

Dorsolateral prefrontal cortex (DLPFC) is the frequently targeted cortical site for cognitive recovery using NIBS techniques. Additionally, the DLPFC has been associated with executive functions, as per a recent organizing principle that distinguishes cognitive processes from affective/reward-related processes [63]. A previous study [64] showed that DLPFC stimulation can amplify DMN node deactivations and enhance high cognitive demand processing. It can maximize therapeutic efficacy through the integration of HF-rTMS stimulation for unleashing the potential of neuronal recovery through enhanced excitability of ipsilesional hemisphere. Thus, DLPFC is favored selection for cognition recovery.

Further, stimulating the motor cortex has been found to improve ADL function. Augmenting motor cortical excitability in the hemisphere affected by stroke is a crucial prerequisite for neural plasticity. This allows the remaining neurons to reorganize and adapt in response to treatment feedback. As a result, motor cortex (M1) stimulation has shown additional benefits across multiple domains, including enhanced ADL capacity and alleviation of cognitive dysfunction [65].

The superior temporal gyrus (STG) is located in the upper part of the temporal lobe and plays a role in both verbal and non-verbal communication. The right anterior STG is involved in processing object- and space-related information. The left posterior STG is responsible for language processing, auditory short-term memory, and the perception and production of speech [66, 67]. Therefore, this region may contribute to the recovery of executive function.

Anatomical-clinical data suggests that posterior parietal damage is the most common relevant anatomical area for USN. Meanwhile, USN is associated with decreased arousal network activity and an imbalance of cortico-subcortical hemispheric connectivity [68]. According to Maurizio Corbetta et al. [69], the phenomenon of neglect is better understood as a result of impairments in distributed cortical networks responsible for attention control, rather than being solely attributed to structural damage in specific brain regions. Overall, PPC stimulation still has significant effects in improving USN.

c-tDCS and other brain regions not mentioned did not show prominent priority in cognition and living function, maybe more extensive clinical trials with larger sample sizes are required to achieve solid evidence.

Taking into account the aforementioned factors, the combination of an appropriate brain region with rTMS has the potential to yield more favorable therapeutic outcomes. In addition, the restoration of entire brain networks plays a crucial role in the rehabilitation journey of stroke patients.

### Strengths and limitations

To the best of our knowledge, this is the first NMA to rank the efficacy of NIBS modes and targeted brains for function in cognition and daily living of patients with stroke. Additionally, evidence-based clinical decision-making can be enhanced by utilizing NMA, which combines direct and indirect comparisons of trials. This approach provides ranked results that indicate the relative efficacy of each intervention type, aiding in informed decision-making. Moreover, specific studies that primarily focus on enhancing motor function have been deemed valuable additions to NMA in terms of statistical integrity, as they also offer potential benefits for cognition and ADL. Overall, the primary goal of this NMA is to offer improved rehabilitation strategies for patients and provide clinicians with enhanced decision support.

Notwithstanding, our study does have a few limitations. First, part of NIBS modes was not included in the current NMA, which may have an impact on the completeness of the findings. However, the inclusion has covered common clinical treatments and explains and guides current clinical applications. Second, the assessment of outcomes in our study involved the utilization of different cognition scales, which introduced subjectivity and potentially increased heterogeneity. To mitigate this bias, we specifically selected RCTs that incorporated blinding designs. Additionally, we included as many relevant scales as possible to ensure comprehensive results. However, given the diversity of assessments, it was not feasible to include all interventions in the partial ranking. Therefore, the results of our study should only be applied to the interventions included in the analysis.

## Conclusions

HF-rTMS is recognized as the superior NIBS intervention for ameliorating overall cognitive impairment. In contrast, Dual-rTMS has exhibited greater effectiveness in improving ADL functioning, while LF-rTMS has demonstrated advantages in enhancing memory and alleviating USN. For enhancing global cognition, DLPFC is highly recommended, whereas the motor cortex (M1) can also be beneficial for improving ADL function. Additionally, PPC is a recommended option for alleviating USN.

### Supplementary Information

Below is the link to the electronic supplementary material.

Supplementary file1 (DOCX 5553 KB)

## Data Availability

The authors confirm that the data supporting the findings of this study are available within the article and its supplementary materials.
