# Supplementary material for: Comparative efficacy of non-invasive brain stimulation for post-stroke cognitive impairment: a network meta-analysis
Source: Aging Clin Exp Res. 2024 Feb 12;36(1):37. doi: 10.1007/s40520-023-02662-x (PMC10861650; doi:10.1007/s40520-023-02662-x)
Supplement: Supplementary file 1 — Supplementary file1 (DOCX 5553 KB) [file 40520_2023_2662_MOESM1_ESM.docx]

**Comparative efficacy of non-invasive brain stimulation for post-stroke cognitive impairment: A network meta-analysis**

**Contents of Supplementary Materials**

| **Contents** |  | **Page#** |
| --- | --- | --- |
| **Supplementary Appendix 1** | PRISMA-NMA checklist | 2-5 |
| **Supplementary Appendix 2** | Search strategy | 6-10 |
| **Supplementary Appendix 3** | Introduction of scales mentioned | 10-11 |
| **Supplementary Appendix 4** | Adverse events and serious adverse events of included randomized controlled trials | 12-13 |
| **Supplementary Table 1** | Intervention-related features of the included studies | 14-33 |
| **Supplementary Figure 1** | Network geometry of interventions across cognition function in the short-term assessment | 34-36 |
| **Supplementary Figure 2** | Forest plots of network meta-analyses compared with placebo across various cognition domains, pooling the effects of NIBS modalities (number of trials ≥2) | 37-39 |
| **Supplementary Table 2** | Rankings of different interventions | 40-41 |
| **Supplementary Figure 3** | Risk of bias summary of included studies | 42 |
| **Supplementary Figure 4** | Proportion of risk levels of bias in each domain | 43 |
| **Supplementary Figure 5** | Evaluation of the inconsistency | 44-45 |
| **Supplementary References** | References of 26 included trials | 46-48 |

**Supplementary Appendix 1** PRISMA-NMA checklist


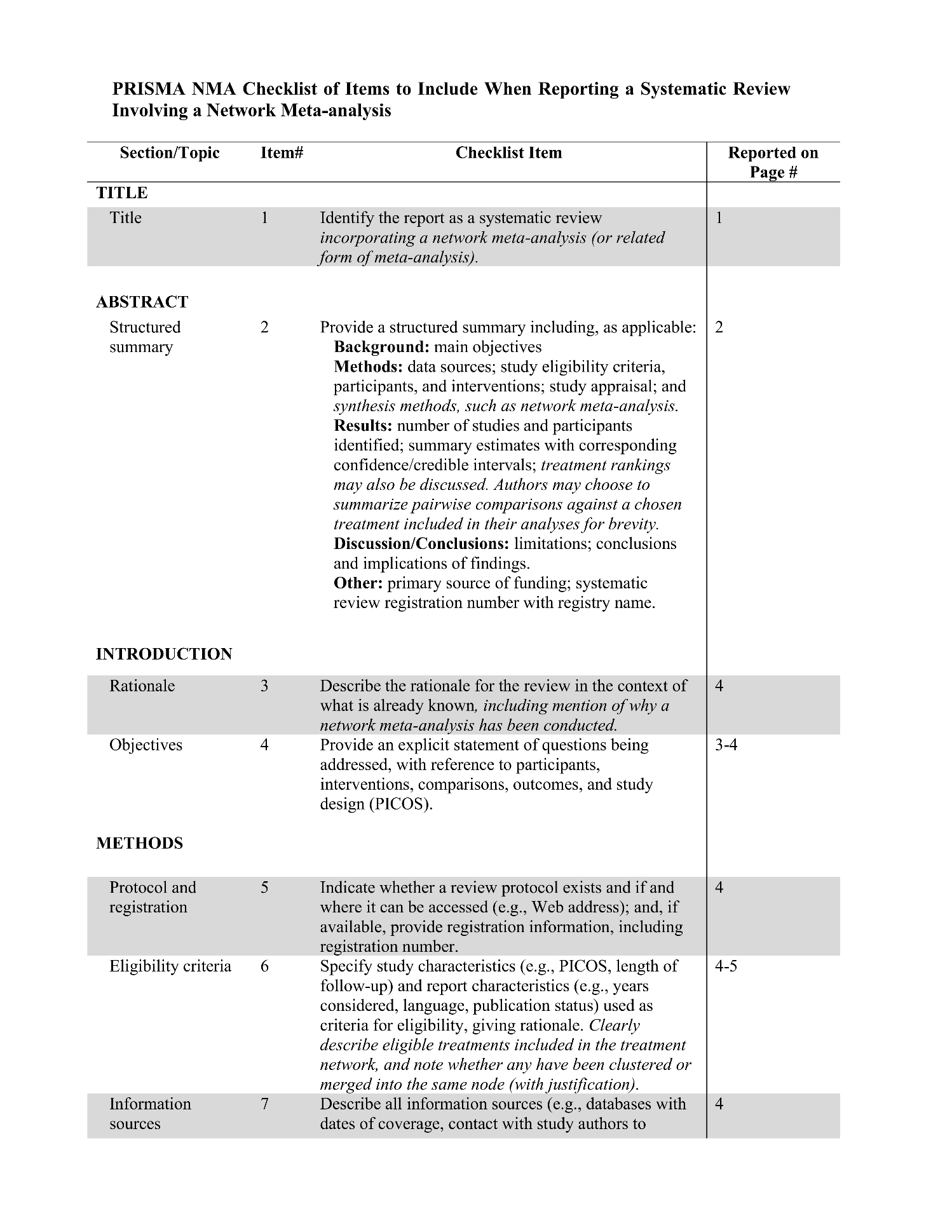

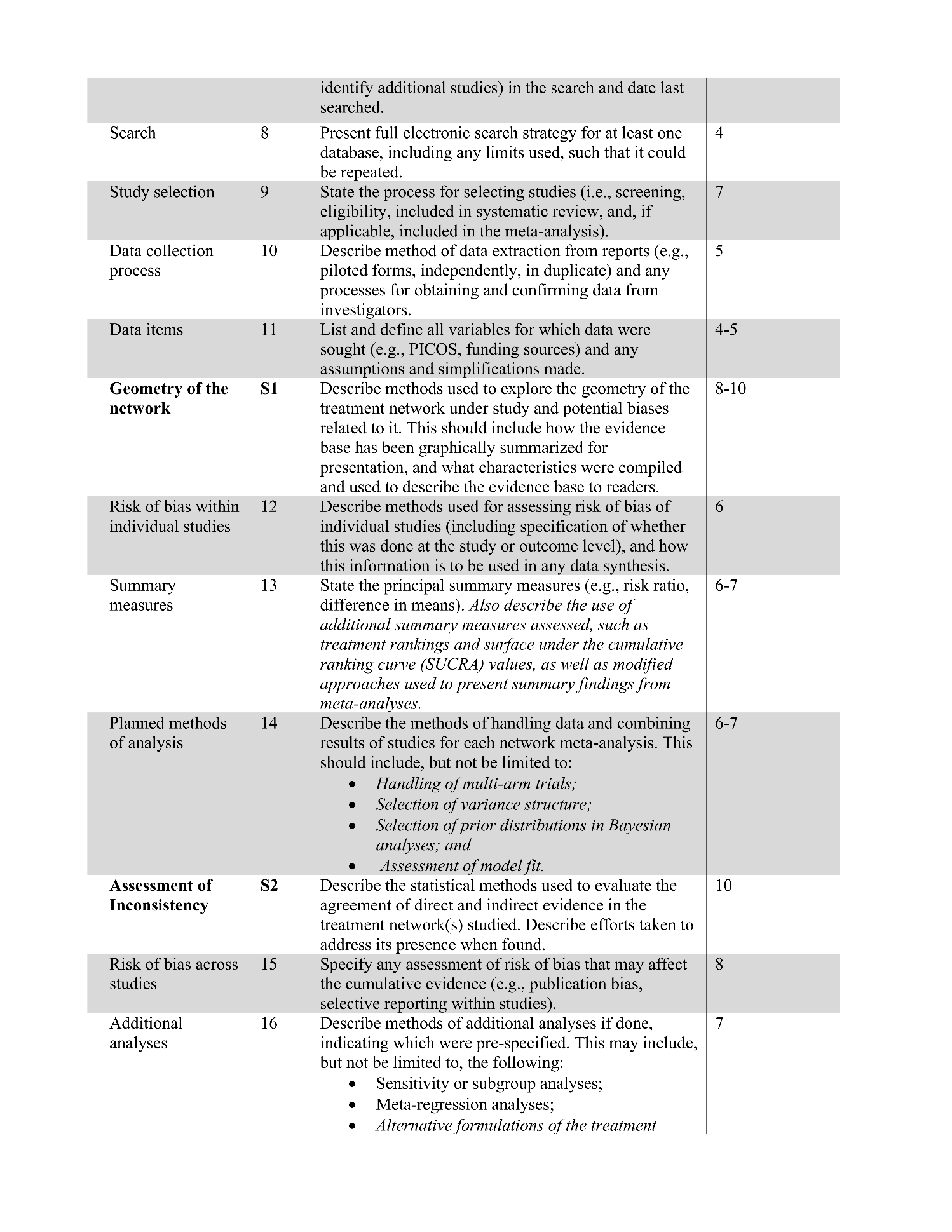

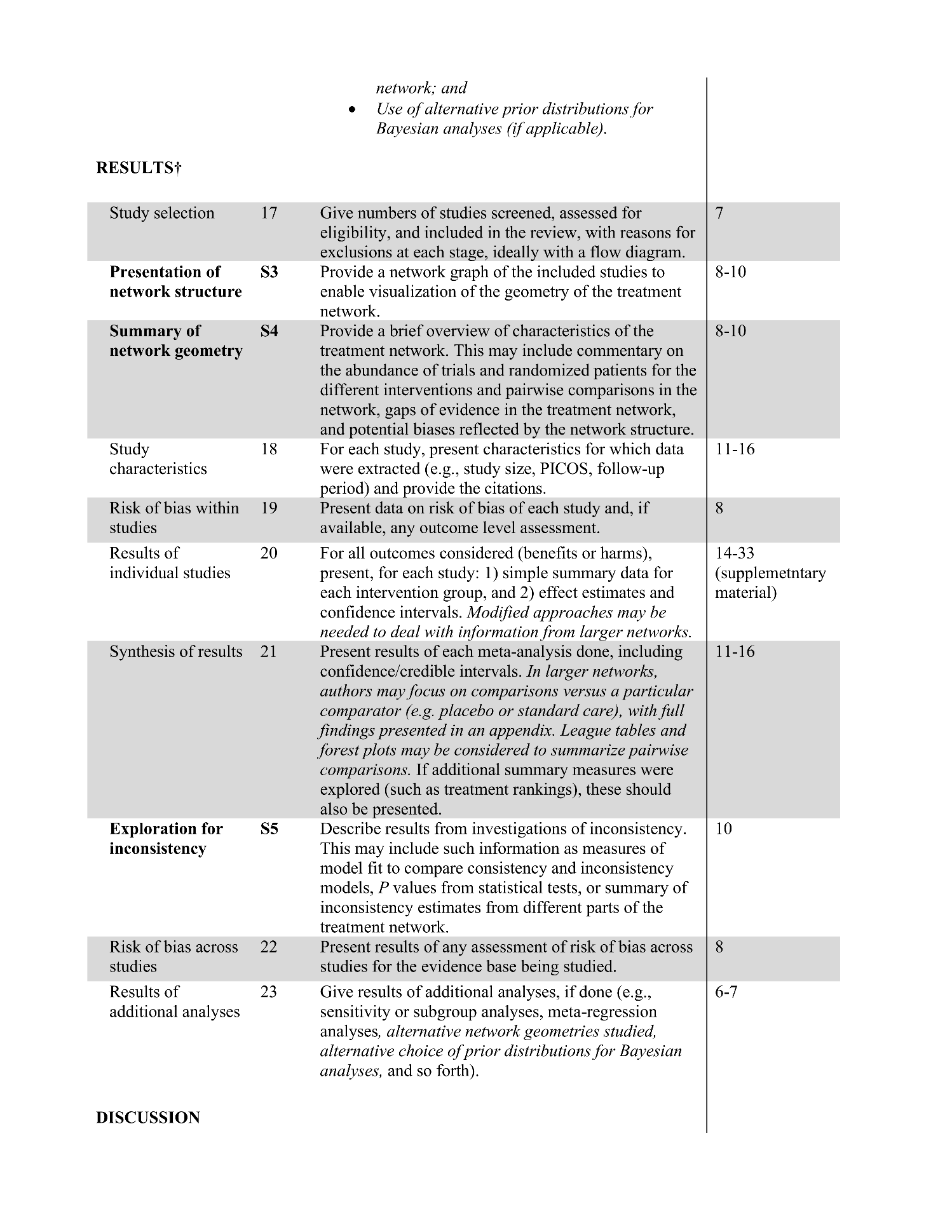

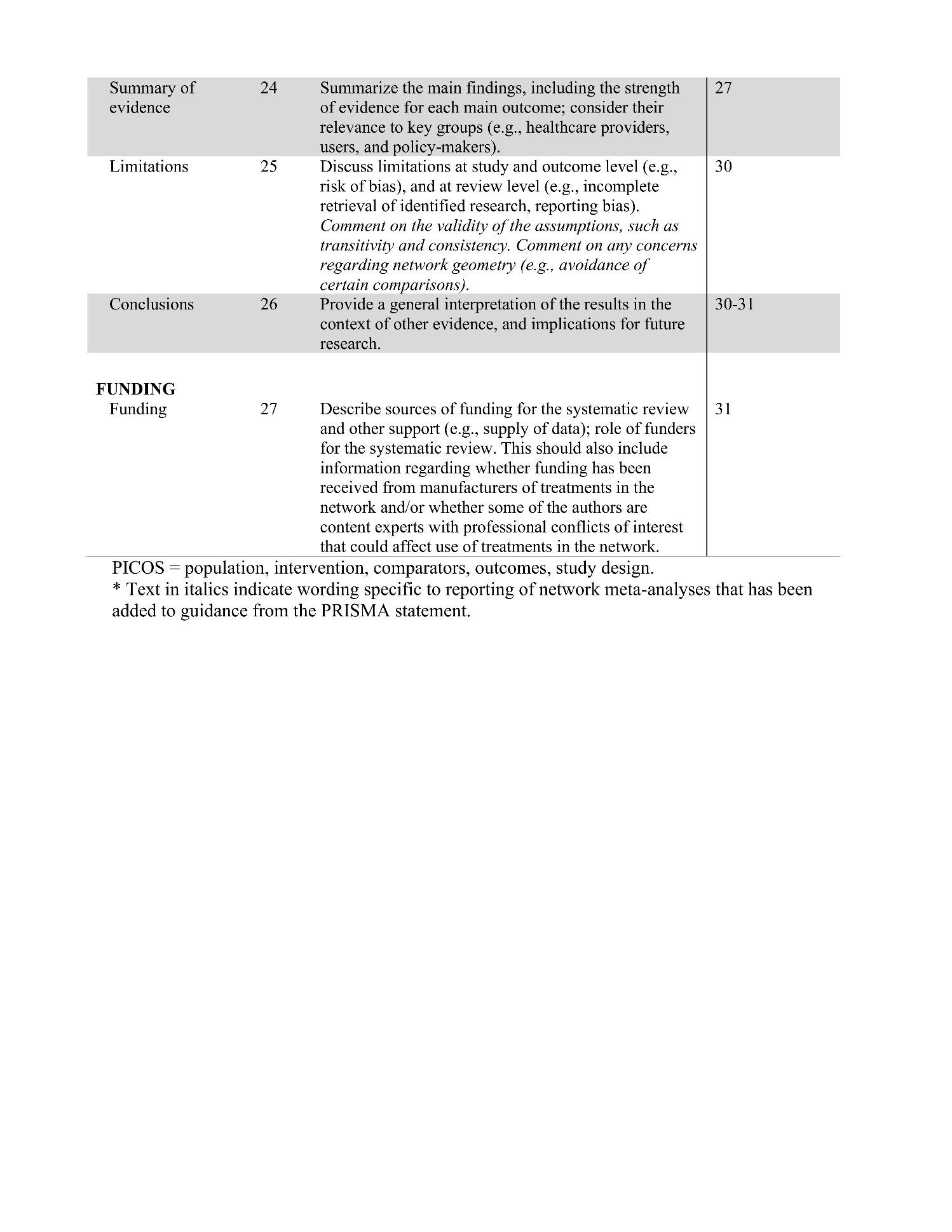


**Supplementary Appendix 2** Search strategy

| **Pubmed** **(searched on June 21, 2022)** | |
| --- | --- |
| #1 | stroke [MeSH Terms] |
| #2 | "Acute Cerebrovascular Accident"[Title/Abstract] OR "Acute Cerebrovascular Accidents"[Title/Abstract] OR "acute cerebrovascular lesion"[Title/Abstract] OR "acute focal cerebral vasculopathy"[Title/Abstract] OR "acute stroke"[Title/Abstract] OR "Acute Strokes"[Title/Abstract] OR "apoplectic stroke"[Title/Abstract] OR "apoplexia"[Title/Abstract] OR "apoplexy"[Title/Abstract] OR "brain accident"[Title/Abstract] OR "brain attack"[Title/Abstract] OR "brain blood flow disturbance"[Title/Abstract] OR "brain insult"[Title/Abstract] OR "brain insultus"[Title/Abstract] OR "brain vascular accident"[Title/Abstract] OR "Brain Vascular Accidents"[Title/Abstract] OR "brainstem stroke"[Title/Abstract] OR "cardioembolic stroke"[Title/Abstract] OR "cerebral apoplexia"[Title/Abstract] OR "cerebral insult"[Title/Abstract] OR "cerebral stroke"[Title/Abstract] OR "Cerebral Strokes"[Title/Abstract] OR "cerebral vascular accident"[Title/Abstract] OR "cerebral vascular insufficiency"[Title/Abstract] OR "cerebro vascular accident"[Title/Abstract] OR "cerebrovascular accident"[Title/Abstract] OR "Cerebrovascular Accidents"[Title/Abstract] OR "Cerebrovascular Apoplexy"[Title/Abstract] OR "cerebrovascular arrest"[Title/Abstract] OR "cerebrovascular failure"[Title/Abstract] OR "cerebrovascular injury"[Title/Abstract] OR "cerebrovascular insufficiency"[Title/Abstract] OR "cerebrovascular insult"[Title/Abstract] OR "Cerebrovascular Stroke"[Title/Abstract] OR "Cerebrovascular Strokes"[Title/Abstract] OR "cerebrum vascular accident"[Title/Abstract] OR "cryptogenic stroke"[Title/Abstract] OR "CVA"[Title/Abstract] OR "experimental stroke"[Title/Abstract] OR "insultus cerebralis"[Title/Abstract] OR "ischaemic seizure"[Title/Abstract] OR "ischemic seizure"[Title/Abstract] OR "ischemic stroke"[Title/Abstract] OR "lacunar stroke"[Title/Abstract] OR "stroke"[Title/Abstract] OR "Strokes"[Title/Abstract] OR "thrombotic stroke"[Title/Abstract] |
| #3 | (Transcranial Magnetic Stimulation [MeSH Terms]) OR (transcranial direct current stimulation [MeSH Terms]) |
| #4 | "Transcranial Magnetic Stimulation"[Title/Abstract] OR "repetitive transcranial magnetic stimulation"[Title/Abstract] OR "Transcranial Magnetic Stimulations"[Title/Abstract] OR "transcranial direct current stimulation"[Title/Abstract] OR "Anodal Stimulation tDCS"[Title/Abstract] OR "Anodal Stimulation tDCSs"[Title/Abstract] OR "Anodal Stimulation Transcranial Direct Current Stimulation"[Title/Abstract] OR "Cathodal Stimulation tDCS"[Title/Abstract] OR "Cathodal Stimulation tDCSs"[Title/Abstract] OR "Cathodal Stimulation Transcranial Direct Current Stimulation"[Title/Abstract] OR "Repetitive Transcranial Electrical Stimulation"[Title/Abstract] OR "tDCS"[Title/Abstract] OR "Transcranial Alternating Current Stimulation"[Title/Abstract] OR "Transcranial Electrical Stimulation"[Title/Abstract] OR "Transcranial Electrical Stimulations"[Title/Abstract] OR "Transcranial Random Noise Stimulation"[Title/Abstract] |
| #5 | cognition [MeSH Terms] |
| #6 | "attention" OR "attribution " OR "Awareness" OR "Cognitions" OR "cognitive accessibility" OR "cognitive appraisal" OR "cognitive balance" OR "cognitive bias" OR "cognitive dissonance" OR "cognitive flexibility" OR "cognitive function" OR "Cognitive Functions" OR "cognitive reserve" OR "cognitive rumination" OR "cognitive structure" OR "cognitive symptoms" OR "cognitive task" OR "cognitive thinking" OR "Comprehension" OR "confusion " OR "Consciousness" OR "Dreams" OR "empowerment" OR "executive function" OR "externalization" OR "fantasy" OR "guided imagery" OR "imagination" OR "internalization " OR "intuition" OR "learning" OR "memory" OR "mental capacity" OR "mental development" OR "mental performance" OR "mental representation" OR "mentalization" OR "metacognition" OR "neurobehavioural manifestations" OR "numerical cognition" OR "orientation" OR "perception" OR "social cognition" OR "theory of mind" OR "thinking" OR "volition" |
| #7 | (#1 OR #2) AND (#3 OR #4) AND (#5 OR #6) |

| **Embase (searched on June 21, 2022)** | |
| --- | --- |
| #1 | 'cerebrovascular accident'/exp |
| #2 | 'acute cerebrovascular accident':ti,ab,kw OR 'acute cerebrovascular accidents':ti,ab,kw OR 'acute cerebrovascular lesion':ti,ab,kw OR 'acute focal cerebral vasculopathy':ti,ab,kw OR 'acute stroke':ti,ab,kw OR 'acute strokes':ti,ab,kw OR 'apoplectic stroke':ti,ab,kw OR 'apoplexia':ti,ab,kw OR 'apoplexy':ti,ab,kw OR 'brain accident':ti,ab,kw OR 'brain attack':ti,ab,kw OR 'brain blood flow disturbance':ti,ab,kw OR 'brain insult':ti,ab,kw OR 'brain insultus':ti,ab,kw OR 'brain vascular accident':ti,ab,kw OR 'brain vascular accidents':ti,ab,kw OR 'brainstem stroke':ti,ab,kw OR 'cardioembolic stroke':ti,ab,kw OR 'cerebral apoplexia':ti,ab,kw OR 'cerebral insult':ti,ab,kw OR 'cerebral stroke':ti,ab,kw OR 'cerebral strokes':ti,ab,kw OR 'cerebral vascular accident':ti,ab,kw OR 'cerebral vascular insufficiency':ti,ab,kw OR 'cerebro vascular accident':ti,ab,kw OR 'cerebrovascular accident':ti,ab,kw OR 'cerebrovascular accidents':ti,ab,kw OR 'cerebrovascular apoplexy':ti,ab,kw OR 'cerebrovascular arrest':ti,ab,kw OR 'cerebrovascular failure':ti,ab,kw OR 'cerebrovascular injury':ti,ab,kw OR 'cerebrovascular insufficiency':ti,ab,kw OR 'cerebrovascular insult':ti,ab,kw OR 'cerebrovascular stroke':ti,ab,kw OR 'cerebrovascular strokes':ti,ab,kw OR 'cerebrum vascular accident':ti,ab,kw OR 'cryptogenic stroke':ti,ab,kw OR 'cva':ti,ab,kw OR 'experimental stroke':ti,ab,kw OR 'insultus cerebralis':ti,ab,kw OR 'ischaemic seizure':ti,ab,kw OR 'ischemic seizure':ti,ab,kw OR 'ischemic stroke':ti,ab,kw OR 'lacunar stroke':ti,ab,kw OR 'stroke':ti,ab,kw OR 'strokes':ti,ab,kw OR 'thrombotic stroke':ti,ab,kw |
| #3 | 'transcranial magnetic stimulation'/exp OR 'transcranial direct current stimulation'/exp |
| #4 | 'transcranial magnetic stimulation':ti,ab,kw OR 'repetitive transcranial magnetic stimulation':ti,ab,kw OR 'transcranial magnetic stimulations':ti,ab,kw OR 'transcranial direct current stimulation':ti,ab,kw OR 'anodal stimulation tdcs':ti,ab,kw OR 'anodal stimulation tdcss':ti,ab,kw OR 'anodal stimulation transcranial direct current stimulation':ti,ab,kw OR 'cathodal stimulation tdcs':ti,ab,kw OR 'cathodal stimulation tdcss':ti,ab,kw OR 'cathodal stimulation transcranial direct current stimulation':ti,ab,kw OR 'repetitive transcranial electrical stimulation':ti,ab,kw OR 'tdcs':ti,ab,kw OR 'transcranial alternating current stimulation':ti,ab,kw OR 'transcranial electrical stimulation':ti,ab,kw OR 'transcranial electrical stimulations':ti,ab,kw OR 'transcranial random noise stimulation':ti,ab,kw |
| #5 | 'cognition'/exp |
| #6 | 'attention':ti,ab,kw OR 'attribution':ti,ab,kw OR 'awareness':ti,ab,kw OR 'cognitions':ti,ab,kw OR 'cognitive accessibility':ti,ab,kw OR 'cognitive appraisal':ti,ab,kw OR 'cognitive balance':ti,ab,kw OR 'cognitive bias':ti,ab,kw OR 'cognitive dissonance':ti,ab,kw OR 'cognitive flexibility':ti,ab,kw OR 'cognitive function':ti,ab,kw OR 'cognitive functions':ti,ab,kw OR 'cognitive reserve':ti,ab,kw OR 'cognitive rumination':ti,ab,kw OR 'cognitive structure':ti,ab,kw OR 'cognitive symptoms':ti,ab,kw OR 'cognitive task':ti,ab,kw OR 'cognitive thinking':ti,ab,kw OR 'comprehension':ti,ab,kw OR 'confusion':ti,ab,kw OR 'consciousness':ti,ab,kw OR 'dreams':ti,ab,kw OR 'empowerment':ti,ab,kw OR 'executive function':ti,ab,kw OR 'externalization':ti,ab,kw OR 'fantasy':ti,ab,kw OR 'guided imagery':ti,ab,kw OR 'imagination':ti,ab,kw OR 'internalization':ti,ab,kw OR 'intuition':ti,ab,kw OR 'learning':ti,ab,kw OR 'memory':ti,ab,kw OR 'mental capacity':ti,ab,kw OR 'mental development':ti,ab,kw OR 'mental performance':ti,ab,kw OR 'mental representation':ti,ab,kw OR 'mentalization':ti,ab,kw OR 'metacognition':ti,ab,kw OR 'neurobehavioural manifestations':ti,ab,kw OR 'numerical cognition':ti,ab,kw OR 'orientation':ti,ab,kw OR 'perception':ti,ab,kw OR 'social cognition':ti,ab,kw OR 'theory of mind':ti,ab,kw OR 'thinking':ti,ab,kw OR 'volition':ti,ab,kw |
| #7 | (#1 OR #2) AND (#3 OR #4) AND (#5 OR #6) |

| **Cochrane Central Register of Controlled Trials (CENTRAL) (searched on June 21, 2022)** | |
| --- | --- |
| #1 | ('Acute Cerebrovascular Accident' OR 'Acute Cerebrovascular Accidents' OR 'acute cerebrovascular lesion' OR 'acute focal cerebral vasculopathy' OR 'acute stroke' OR 'Acute Strokes' OR 'apoplectic stroke' OR 'apoplexia' OR 'apoplexy' OR 'brain accident' OR 'brain attack' OR 'brain blood flow disturbance' OR 'brain insult' OR 'brain insultus' OR 'brain vascular accident' OR 'Brain Vascular Accidents' OR 'brainstem stroke' OR 'cardioembolic stroke' OR 'cerebral apoplexia' OR 'cerebral insult' OR 'cerebral stroke' OR 'Cerebral Strokes' OR 'cerebral vascular accident' OR 'cerebral vascular insufficiency' OR 'cerebro vascular accident' OR 'cerebrovascular accident' OR 'Cerebrovascular Accidents' OR 'Cerebrovascular Apoplexy' OR 'cerebrovascular arrest' OR 'cerebrovascular failure' OR 'cerebrovascular injury' OR 'cerebrovascular insufficiency' OR 'cerebrovascular insult' OR 'Cerebrovascular Stroke' OR 'Cerebrovascular Strokes' OR 'cerebrum vascular accident' OR 'cryptogenic stroke' OR 'CVA' OR 'experimental stroke' OR 'insultus cerebralis' OR 'ischaemic seizure' OR 'ischemic seizure' OR 'ischemic stroke' OR 'lacunar stroke' OR 'stroke' OR 'Strokes' OR 'thrombotic stroke'):ti,ab,kw |
| #2 | MeSH descriptor: [Stroke] explode all trees |
| #3 | ('Transcranial Magnetic Stimulation' OR 'repetitive transcranial magnetic stimulation' OR 'Transcranial Magnetic Stimulations' OR 'transcranial direct current stimulation' OR 'Anodal Stimulation tDCS' OR 'Anodal Stimulation tDCSs' OR 'Anodal Stimulation Transcranial Direct Current Stimulation' OR 'Cathodal Stimulation tDCS' OR 'Cathodal Stimulation tDCSs' OR 'Cathodal Stimulation Transcranial Direct Current Stimulation' OR 'Repetitive Transcranial Electrical Stimulation' OR 'tDCS' OR 'Transcranial Alternating Current Stimulation' OR 'Transcranial Electrical Stimulation' OR 'Transcranial Electrical Stimulations' OR 'Transcranial Random Noise Stimulation'):ti,ab,kw |
| #4 | MeSH descriptor: [Transcranial Direct Current Stimulation] explode all trees |
| #5 | MeSH descriptor: [Transcranial Magnetic Stimulation] explode all trees |
| #6 | MeSH descriptor: [Cognition] explode all trees |
| #7 | ('attention' OR 'attribution ' OR 'Awareness' OR 'Cognitions' OR 'cognitive accessibility' OR 'cognitive appraisal' OR 'cognitive balance' OR 'cognitive bias' OR 'cognitive dissonance' OR 'cognitive flexibility' OR 'cognitive function' OR 'Cognitive Functions' OR 'cognitive reserve' OR 'cognitive rumination' OR 'cognitive structure' OR 'cognitive symptoms' OR 'cognitive task' OR 'cognitive thinking' OR 'Comprehension' OR 'confusion ' OR 'Consciousness' OR 'Dreams' OR 'empowerment' OR 'executive function' OR 'externalization' OR 'fantasy' OR 'guided imagery' OR 'imagination' OR 'internalization ' OR 'intuition' OR 'learning' OR 'memory' OR 'mental capacity' OR 'mental development' OR 'mental performance' OR 'mental representation' OR 'mentalization' OR 'metacognition' OR 'neurobehavioural manifestations' OR 'numerical cognition' OR 'orientation' OR 'perception' OR 'social cognition' OR 'theory of mind' OR 'thinking' OR 'volition'):ti,ab,kw |
| #8 | (#1 OR #2) AND (#3 OR #4 OR #5) AND (#6 OR #7) |

**Supplementary Appendix 3** Introduction of scales mentioned

| **Scale** | **Full name** | **Represented domain** | **Detail** |
| --- | --- | --- | --- |
| **MMSE** | Mini-Mental State Examination | Global cognition severity | For fast and uncomplicated cognitive screening. |
| **MoCA** | Montreal Cognitive Assessment |  | A tracking tool for post-stroke patients with cognition deficit. |
| **BI** | Barthel Index | ADL (activities of daily living) | Tools for assessing basic activities of daily living. |
| **MBI** | Modified Barthel Index |  |  |
| **FIM** | Functional Independence Measure |  | It is more detailed, precise and sensitive than the Barthel index in the way it reflects the level of disability or the amount of help needed, and is a powerful indicator of the effectiveness of rehabilitation. |
| **NIHSS** | National Institutes of Health Stroke Scale | Global stroke severity | A higher score indicates a more severe stroke and is positively correlated with the volume of brain damage caused by stroke. |
| **MVPT** | Motor Free Visual Perception Test | USN (unilateral spatial neglect) | A measure of the visual perceptual process. |
| **CBS** | Catherine Bergego Scale |  | A functional scale to score depending on the observation of the patient with USN in daily living situations. |
| **LBT** | Line Bisection Test |  | Two scales of nine behavioral tests which simulate daily living activities to draw closer to the real impact of the condition. |
| **SCT** | Star Cancelation Test |  |  |
| **TMT** | Trail Making Test | Executive function | An indicator of visual scanning, graphomotor speed, and executive function. |
| **RBMT** | Rivermead Behavioural Memory Test | Memory | Assessing everyday memory using tasks that mimic everyday challenges. |

MMSE and MoCA are preferred measurements of global cognition severity, BI, MBI, FIM and NIHSS are selected because they are better in representing ADL and severity of stroke. In terms of other domains of cognition, RBMT assesses memory function. TMT is used as an indicator of executive function. MVPT, LBT, SCT, and CBS have been selected to represent USN.

**Supplementary Appendix 4** Adverse events and serious adverse events of included randomized controlled trials

| **Study(year)** | **Adverse events (Symptoms and number of reported participants)** | **Dropout** |
| --- | --- | --- |
| ***TMS*** |  |  |
| Qingmei Chen et al. (2021) [1] | Temporary headaches (3), tingling (2). | NM |
| Hong Li et al. (2021) [2] | NM | 2 in follow-up (rTMS group);  3 in follow-up (sham group) |
| Fangzhou Yu et al. (2021) [3] | NM | NM |
| Mingyu Yin et al. (2020) [4] | NM | NM |
| Yamei Li et al. (2020) [5] | Dizziness or headache(several) | None |
| Yuanwen Liu et al. (2020) [6] | None | 2 in follow-up (rTMS group);  2 in follow-up (sham group) |
| Shole Vatanparasti et al. (2019) [7] | None | NM |
| Thomas Nyffeler et al. (2019) [8] | None | Some in follow-up (cTBS group) |
| Sang Beom Kim et al. (2018) [9] | NM | 9 in follow-up (in all) |
| Ayhan Askin et al. (2017) [10] | None | NM |
| Ko Un Kim et al. (2017) [11] | NM | NM |
| Koichi Hosomi et al. (2016) [12] | None | NM |
| Haitao Lu et al. (2015) [13] | Headache (1), dizziness (1), transient headache (1). | None |
| Wei Yang et al. (2015) [14] | NM | None |
| Hyun Gyu Cha et al. (2015) [15] | NM | 1 in follow-up (rTMS group);  1 in follow-up (sham group) |
| Bo Ryun Kim et al. (2013) [16] | None | 6 in follow-up (in all) |
| Dario Cazzoli et al. (2012) [17] | None | NM |
| G. Koch et al. (2012) [18] | None | 1 in study (cTBS group); 1 in study (sham group) |
| ***tDCS*** |  |  |
| Danielle De S. Boasquevisque et al. (2021) [19] | NM | 1 in baseline ,2 in follow-up (tDCS group); 1 in baseline, 1 in follow-up (sham group) |
| Hussien Ahmed Shaker et al. (2018) [20] | NM | NM |
| Hosseinzadeh et al. (2018) [21] | None | NM |
| You Gyoung Yi et al. (2016) [22] | None | 2 in follow-up (in all) |
| Ko Un Kim et al. (2016) [23] | NM | NM |
| Gi Jeong Yun et al. (2015) [24] | NM | NM |
| See Hyun Park et al. (2013) [25] | Pricking sensation (several). | NM |
| Hyuk Sunwoo et al. (2013) [26] | None | NM |

NM, Not mentioned.

**Supplementary Table 1** Intervention-related features of the included studies

| **Study(year)** | **Group** | **Parameter** | **Sessions** | **Targets** | **Assessments** | **Results**  **(For each group)** | **Rehabilitation (For all patients)** |
| --- | --- | --- | --- | --- | --- | --- | --- |
| ***TMS*** |  |  |  |  |  |  |  |
| Qingmei Chen et al. (2021) [1] | A-Dual-rTMS-M1 | 10 Hz,90%MT,600 pulses/session | 20 | motor recovery | NIHSS, mRS, FMA, FMA-UL, ADL, MMSE, HAMD | At post-intervention, group A showed greater motor improvements in FMA, FMA-UL, NIHSS, ADL and mRS  values than group B, group C and group D, that were continued for at least 3 months after the completion of the treatment  time. The situation of the M1 did  not employ any balance effect on emotion or cognition. | All patients got the same routine medical treatment and physiotherapy 30 min per day comprised of task-oriented training activities of daily living instruction and individualized motor tasks. The daily sessions were conducted for 5 consecutive days in a week and sustained for four weeks. |
|  | B-HF-rTMS-M1 |  |  |  |  |  |  |
|  | C-LF-rTMS-M1 |  |  |  |  |  |  |
|  | D-Placebo |  |  |  |  |  |  |
| Hong Li et al. (2021) [2] | LF-rTMS-DLPFC | 1 Hz,90%MT,1000 pulses/session | 20 | Thyroid Hormones Level and Cognition | MMSE, MoCA, MBI | Post-treatment MoCA  scores, MBI, and scores for the 4 cognitive domains in both  groups were significantly higher than the respective pre-treatment scores (P <0.05 for all) | Subjects in both groups received basic treatments, including conventional medication, conventional rehabilitation, and cognitive training. The  rTMS group received basic treatment and rTMS treatment, while the sham group received basic treatment and sham stimulation treatment. |
|  | placebo |  |  |  |  |  |  |
| Fangzhou Yu et al. (2021) [3] | HF-rTMS-DLPFC | 10Hz,90%MT,20 pulses/session | 24 | psychological emotions and cognitive and neurological  functions | SAS, SDS, NIHSS, MMSE, BI, SF-36 | Compared with the placebo group, the SAS, SDS, and NIHSS scores in  the HF-rTMS-DLPFC group decreased, while the MMSE and BI scores increased (P < 0.05). After the treatment, the SF-36 scores in the HF-rTMS-DLPFC group were higher than they were in the placebo group (P < 0.05). | After admission, the patients in both groups were administered routine treatment for acute stroke, including anti-platelet aggregation, protection of the brain cells and symptomatic support, and routine functional recovery training. |
|  | placebo |  |  |  |  |  |  |
| Mingyu Yin et al. (2020) [4] | HF-rTMS-DLPFC | 10Hz,80%MT,2000 pulses/session | 20 | cognitive impairment | MoCA,VST,RBMT,MBI | Pairwise comparisons showed that the MoCA score in both groups increased significantly after 2 and 4 weeks (P < 0.05); RBMT score increased significantly for HF-rTMS-DLPFC group after 2 and 4 weeks of treatments (P < 0.001); MBI scores increased significantly after 2 and 4 weeks of treatments for both groups (P < 0.05); the time consumed of VST-C in HF-rTMS-DLPFC group was significantly lower than that in the no-stim placebo group after 4 weeks of treatments (P = 0.03). | After rTMS treatments, patients received a 30-min computer assisted cognitive rehabilitation referring to attention, executive function, memory, calculation, language and visuospatial skills, etc. Therapists were blinded to assignments. Besides, during hospitalization, patients received conventional drug treatments recommended by the 2016 American Heart Association/American Stroke Association recommendation. |
|  | placebo |  |  |  |  |  |  |
| Yamei Li et al. (2020) [5] | HF-rTMS-DLPFC | 5Hz,100%MT,2000 pulses/session | 15 | cognitive impairment | MMSE,MoCA | Cognition improvements were observed both two groups (P < 0.01), while the HF-rTMS-DLPFC group got more significant improvement than  placebo group (P < 0.05). | Routine cognitive training was given to both groups. Cognitive training covered several domains of cognition and performed as follows, memory, attention, orientation, visual and spatial perception, judging and reasoning ability, executive capability. |
|  | placebo |  |  |  |  |  |  |
| Yuanwen Liu et al. (2020) [6] | HF-rTMS-DLPFC | 10Hz,90%MT,700 pulses/session | 20 | performance  of the ADL and  attention function | FIM, TMT-A, DST, DS, MMSE | HF-rTMS-DLPFC group was significantly improved in all assessment  categories compared with the placebo group. (P< 0.05). | All the patients were given comprehensive cognitive training. |
|  | placebo |  |  |  |  |  |  |
| Ayhan Askin et al. (2017) [10] | LF-rTMS-M1 | 1 Hz,90% MT,1200 pulses/session | 10 | upper extremity motor  recovery and functional outcomes | BRS, UE-FMA, BBT, MAS, FIM, MMSE, FAS | There were statistically significant  improvements in all clinical outcome measures except for  the BRS. FIM cognitive scores and MMSE scores  were increased and distal and hand MAS scores  were decreased only in the LF-rTMS-M1 group (p < 0.05). | NM |
|  | placebo |  |  |  |  |  |  |
| Koichi Hosomi et al. (2016) [12] | HF-rTMS-M1 | 5 Hz,90%MT,500 pulses/session | 10 | poststroke upper limb paresis | BS, FMA, NIHSS, FIM | The HF-rTMS-M1 group demonstrated additional improvement in the BS hand score at the last follow-up compared to the  placebo group. The grip power, the NIHSS motor score, and the number of finger taps in  the affected hand improved in the HF-rTMS-M1 group. The BS upper limb scores, the FMA distal upper limb score, the NIHSS  total score, and the FIM motor score showed improvement from baseline at the  earlier time points after the real rTMS. | All patients received regular rehabilitation therapy. |
|  | placebo |  |  |  |  |  |  |
| Haitao Lu et al. (2015) [13] | LF-rTMS-DLPFC | 1 Hz,100%MT,600 pulses/session | 20 | post-stroke dysmnesia | MoCA, LOTCA, RBMT | No difference was observed between the LF-rTMS-DLPFC group and the  placebo group for MoCA, LOTCA, and RBMT.  Three days after treatment, MoCA, LOTCA, and RBMT scores were higher after rTMS. Two months after treatment, RMBT scores in the LF-rTMS-DLPFC  group were higher than in the placebo group, but not MoCA and LOTCA scores. | Aside from the real or sham rTMS treatment, all patients received regular computer-assisted cognitive training for 30 min every day. According to the severity of the cognitive disorder, the training included graphical, verbal and spatial memory. In addition, during and after rTMS treatments, patients received secondary stroke prevention drugs as recommended by the 2008 AHA/ASA recommendation for the prevention of stroke in patients with stroke and transient ischemic attacks. |
|  | placebo |  |  |  |  |  |  |
| Dario Cazzoli et al. (2012) [17] | cTBS-PPC | 3 pulses at 30 Hz, repeated at 6 Hz, for  total 44 sec (801 pulses),100%MT | 8 | spatial neglect | CBS, VT, paper–pencil assessment | The results  showed 37% improvement in the spontaneous everyday behavior of the neglect patients after the repeated application of  cTBS. Remarkably, the improvement persisted for at least 3 weeks after stimulation. | During the study, all patients were also undergoing full neurorehabilitation therapy including 1 h neuropsychological training (visuospatial exploration training, and attention and concentration training), 1 h of occupational therapy and 1h of physiotherapy per day. |
|  | cTBS-PPC |  | 8 |  |  |  |  |
|  | placebo |  |  |  |  |  |  |
| Shole Vatanparasti et al. (2019) [7] | cTBS-PPC | 3pulses at 30 Hz, repeated with an inter burst interval of  100 ms (801 pulses),80%MT | 10 | neglect  recovery | SCT, LBT, Figure Copying Test, Clock  Drawing Task, MRS | The results of the present study showed that, cTBS did not increase the effect of prism adaptation on neglect symptoms in  stroke patients. | NM |
|  | placebo |  |  |  |  |  |  |
| Thomas Nyffeler et al. (2019) [8] | placebo | 3 pulses at 30 Hz, repeated at 6 Hz,for  total 44 sec (801 pulses),100%MT |  | functional outcome and response variability  origins in neglect after stroke | CBS, neglect composite score, FIM, LIMOS | In neglect patients with intact interhemispheric connectivity, cTBS significantly improves and accelerates neglect recovery  and general functional outcome. | All patients received interdisciplinary therapy in our neurorehabilitation clinic. In addition, all neglect patients also received smooth pursuit eye movement training, daily over a period of 3 weeks. |
|  | cTBS-PPC |  | 8 |  |  |  |  |
|  | cTBS-PPC |  | 16 |  |  |  |  |
| G. Koch et al. (2012) [18] | cTBS-PPC | 3-pulse bursts at 50 Hz repeated every 200 msec  for 40 s were delivered at 80% of the active motor threshold  (AMT) over the left PPC (600 pulses) | 20 | hemispatial neglect | line  crossing, letter cancellation, SCT, figure and shape  copying, line bisection, and representational drawing | 2 weeks of cTBS were effective in improving neglect  symptoms as measured by BIT score. BIT scores improved by 16.3% after 2 weeks of cTBS and  22.6% at 1 month follow-up. Hyperexcitability of left hemisphere parieto-frontal circuits  was reduced following treatment with real cTBS. | NM |
|  | placebo |  |  |  |  |  |  |
| Sang Beom Kim et al. (2018) [9] | cTBS-PPC | 0.9 Hz,95%MT,900 pulses/session | 10 | hemispatial  neglect in stroke patients | MVPT-3, LBT, SCT, CBS, MMSE, K-MBI | Two weeks after the therapy, all groups showed significant improvement  in MVPT-3, LBT, SCT, CBS, MMSE, and K-MBI. | Patients in the robot group received additional treatment for hemispatial neglect using a rehabilitation robot (Neuro-X; Apsun Inc., Seoul, Korea) for upper limbs. |
|  | placebo |  |  |  |  |  |  |
| Wei Yang et al. (2015) [14] | LF-rTMS-PPC | 1 Hz,80%MT,656 pulse | 20 | unilateral spatial neglect | SCT, LBT, DTI, FA | SCT and LBT revealed significant  differences in outcomes at the end of treatments and one month after the end of treatments. Importantly, cTBS-PPC group patients displayed the best curative effect, based on  behavioral scoring, at one month after end of the treatments, followed by the 1 Hz group and 10 Hz group. DTI  results showed a significant increase in FA and MD in superior longitudinal fasciculus, superior occipitofrontal  fascicle and inferior fronto-occipital fasciculus on the left side, as well as the capsula external and inferior  frontooccipital fasciculus on the right side, in patients after cTBS. | All patients received routine rehabilitation as follows patients underwent “one on one” Bobath treatment with a physical therapist; the job therapist trained patients for the following daily activities, sitting by the bed, conversion between bed and wheelchair, and eating with tableware; speech therapist conducted speech training for 3–4 h per day, 5 days a week for a total 8 weeks of treatment. |
|  | HF-rTMS-PPC | 10 Hz,80%MT,1000 pulse |  |  |  |  |  |
|  | cTBS-PPC | 3 pulses at 30 Hz, repeated at 5 Hz, (801 pulses),80%MT |  |  |  |  |  |
|  | placebo |  |  |  |  |  |  |
| Bo Ryun Kim et al. (2013) [16] | LF-rTMS-PPC | 1 Hz,90%MT,1200 pulse | 10 | visuospatial neglect in patients with  acute stroke | MVPT, LBT, SCT, CBS, K-MBI | LBT and K-MBI scores were significantly different between 3 groups. In the post hoc analysis, the improvement in the LBT  score in the HF-rTMS-PPC group was statistically significant compared with that in the placebo group  , and the improvements in the K-MBI scores of the HF-rTMS-PPC group and LF-rTMS-PPC group were statistically significant compared  with those in the placebo group. | All participants received conventional rehabilitation treatment, including physical, occupational, and cognitive therapies of the same intensity and duration. In addition, all participants received conventional rehabilitation programs for visuospatial neglect, such as visual tracking, reading and writing, drawing and copying, and puzzles. There were no changes to medications that could affect attention. |
|  | HF-rTMS-PPC | 10 Hz,90%MT,1000 pulse |  |  |  |  |  |
|  | placebo |  |  |  |  |  |  |
| Hyun Gyu Cha et al. (2015) [15] | LF-rTMS-PPC | 1 Hz,90%MT | 20 | unilateral neglect of acute stroke patients | MVPT, LBT, AT, SCT | The LF-rTMS-PPC group showed a significant increase in the MVPT, LBT, AT, and SCT  values compared with the preintervention values (p < 0.05). | All patients received conventional rehabilitation therapy consisted of neurodevelopmental facilitation techniques for 30 minutes per day. |
|  | placebo |  |  |  |  |  |  |
| Ko Un Kim et al. (2017) [11] | LF-rTMS-PPC | 1 Hz,90%MT+5 Hz,90%MT | 20 | depression, visual  perception, and ADL | BDI, MVPT, FIM | The LF-rTMS-PPC group showed  significant improvements in depression, visual perception, and ADLs between week 1 and 4, between week 1 and  8, and between week 4 and 8. | The rehabilitation therapy focused on visuospatial neglect, and was conducted at a difficulty matched to each patient’s abilities. |
|  | placebo |  |  |  |  |  |  |
| ***tDCS*** |  |  |  |  |  |  |  |
| Danielle De S. Boasquevisque et al. (2021) [19] | c-tDCS-M1 | 1 mA,35cm²,20min | 6 | the motor impairment, ADL and spasticity of upper limb, overall neurologic impairment, overall disability, functional independence,  quality of life,  cognition,  sleep | NIHSS, FMA, MAL, MAS, mRS, BI, SIS, MoCA, PSQI | At three months post intervention, the placebo group improved significantly  compared to posttreatment in NIHSS, BI. Also at three months, NIHSS improved significantly in the sham group and worsened significantly in the c-tDCS-M1 group. | Physical therapy was delivered after the end of stimulation with 30-minute exercises focused on the upper limb. |
|  | placebo |  |  |  |  |  |  |
| Hussien Ahmed Shaker et al. (2018) [20] | Dual-tDCS-DLPFC | 2 mA × 30 min | 12 | Attention,  Memory | Computer-based cognitive therapy tool  (attention and concentration, figural  memory, reaction behavior, and logical  reasoning.), FIM | There was a significant improvement in the scores of attention and  concentration, figural memory, logical reasoning, reaction behavior in  both groups. However, the improvement was significantly higher in the  Dual-tDCS-DLPFC group compared to the placebo group. | All patients received cognitive training program. |
|  | placebo |  |  |  |  |  |  |
| Hosseinzadeh et al. (2018) [21] | a-tDCS-STG | 2 mA/35 cm² × 30 min | 12 | movement functions, visual attention and depression | NIHSS, TMT,  the Beck Test | The results show that anodic tDCS  application could lead to positive improvements of  movement (based on NIHSS scores), visual attention (based on the TMT), and depression (based on the Beck Test). | NM |
|  | c-tDCS-STG |  |  |  |  |  |  |
|  | placebo |  |  |  |  |  |  |
| Gi Jeong Yun et al. (2015) [24] | a-tDCS-FTP-L | 2 mA/25 cm² × 30 min | 15 | Cognition,  Attention, WM,  Memory | MMSE,  DS, VS, VerL, VisL, VCPT, ACPT, BI | a-tDCS-FTP-L group improved digit and visual span task and verbal  memory. a-tDCS-FTP-R group improved only verbal memory between pre  and post treatment. a-tDCS-FTP-L group significantly improved verbal  memory compared to the other groups. | NM |
|  | a-tDCS-FTP-R |  |  |  |  |  |  |
|  | placebo |  |  |  |  |  |  |
| See Hyun Park et al. (2013) [25] | Dual-tDCS-DLPFC | 2 mA/25 cm² × 30 min | 18.5 | Cognition  Attention, WM | DS, VS, CPT, MMSE | Dual-tDCS-DLPFC group was significantly improved in auditory and visual  continuous performance compared with placebo group. | The Korean computer-assisted cognitive rehabilitation program was concomitantly applied in all patients. |
|  | placebo |  | 17.8 |  |  |  |  |
| Hyuk Sunwoo et al. (2013) [26] | Dual-tDCS-PPC | 1 mA/25 cm² × 20 min |  | unilateral visuospatial neglect | LBT, SCT | In the LBT, significant improvements were observed after  both in Dual-tDCS-PPC and a-tDCS-PPC group (p < 0.05), but not in placebo group. The SCT did not show any significant change. | NM |
|  | a-tDCS-PPC |  |  |  |  |  |  |
|  | placebo |  |  |  |  |  |  |
| You Gyoung Yi et al. (2016) [22] | a-tDCS-M1 | 2 mA/25 cm² × 30 min | 15 | Neglect Syndrome | MVPT,  LBT, SCT, CBS, K-MBI, FAC | The study results indicated that the facilitatory effect of a-tDCS applied over the right PPC, and  the inhibitory effect of c-tDCS applied over the left PPC, improved symptoms of visuospatial neglect., but it might not  lead to improvements in ADL function and gait function. | NM |
|  | Dual-tDCS-PPC |  |  |  |  |  |  |
|  | placebo |  |  |  |  |  |  |
| Ko Un Kim et al. (2016) [23] | a-tDCS-PPC | 1 mA/24 cm² × 20 min | 30 | visual perception function and performance  capability of ADL | MVPT, FIM | Both groups improved in visual perception function and in performance of ADL.  Although there was no significant difference between the two groups, the a-tDCS-PPC group exhibited higher scores. | For all patients, traditional occupational therapy treatment were applied. |
|  | placebo |  |  |  |  |  |  |

NM, Not mentioned.; HF-,high frequency; LF-,low frequency; cTBS, continuous theta burst stimulation; a-, anodal; c-, cathodal; DLPFC, dorsolateral prefrontal cortex; STG, superior temporal gyrus; FTP, fronto-temporal region; PPC, posterior parietal cortex; M1, primary motor cortex; ADL, activities of daily living; rTMS, repetitive transcranial magnetic stimulation; tDCS, transcranial direct current stimulation; FMA, Fugl-Meyer Assessment; FMA-UL, upper limb score of Fugl–Meyer Assessment; mRS, modified Rankin Scale; MMSE, Mini Mental State Examination; HAMD, Hamilton Depression Scale; SAS, The self-rating anxiety scale; SDS, the self-rating depression scale; SF-36, the quality of life scale; BRS, Brunnstrom Recovery Stages; UE-FMA, upper extremity Fugl–Meyer Assessment; BBT, Box and Block test; MAS, Modified Ashworth Scale; FAS, Functional Ambulation Scale; BS, Brunnstrom stage; CBS, Catherine Bergego Scale; VT, Vienna Test System; SCT, Star Cancellation Test; LBT, Line Bisection Task; LIMOS, Lucerne ICF-based Multidisciplinary Observation Scale; MVPT, Motor-Free Visual Perception Test; K-MBI, the Korean version of Modified Barthel Index; DTI, diffusion-tensor imaging; FA, fractional anisotropy; AT, Albert Test; SIS, Stroke Impact Scale; MAL, Motor Activity Log; SIS, Stroke Impact Scale; PSQI, Pittsburgh Sleep Quality Index; FAC, Functional Ambulation Classification; ACPT, Auditory continuous performance test; BDI, Beck’s Depression Inventory; BI, Barthel Index; MBI, Modified Barthel Index; CPT, continuous performance test; DS, Digit Span; DST, Digit Symbol Test; FIM, Functional Independence Measure; LOTCA, Loewenstein Occupational Therapy of Cognitive Assessment; MoCA, Montreal Cognitive Assessment; MT, Motor threshold; NIHSS, National Institutes of Health Stroke Scale; RBMT, Rivermead Behavior Memory Test; TMT, Trail Making Test; ToL, Tower of London test; VCPT, visual continuous performance test; VerL, verbal learning test, VisL; visual learning test; VS, Visual span; VST, Victoria Stroop Test; WM, Working Memory; L, left; R, right.

**
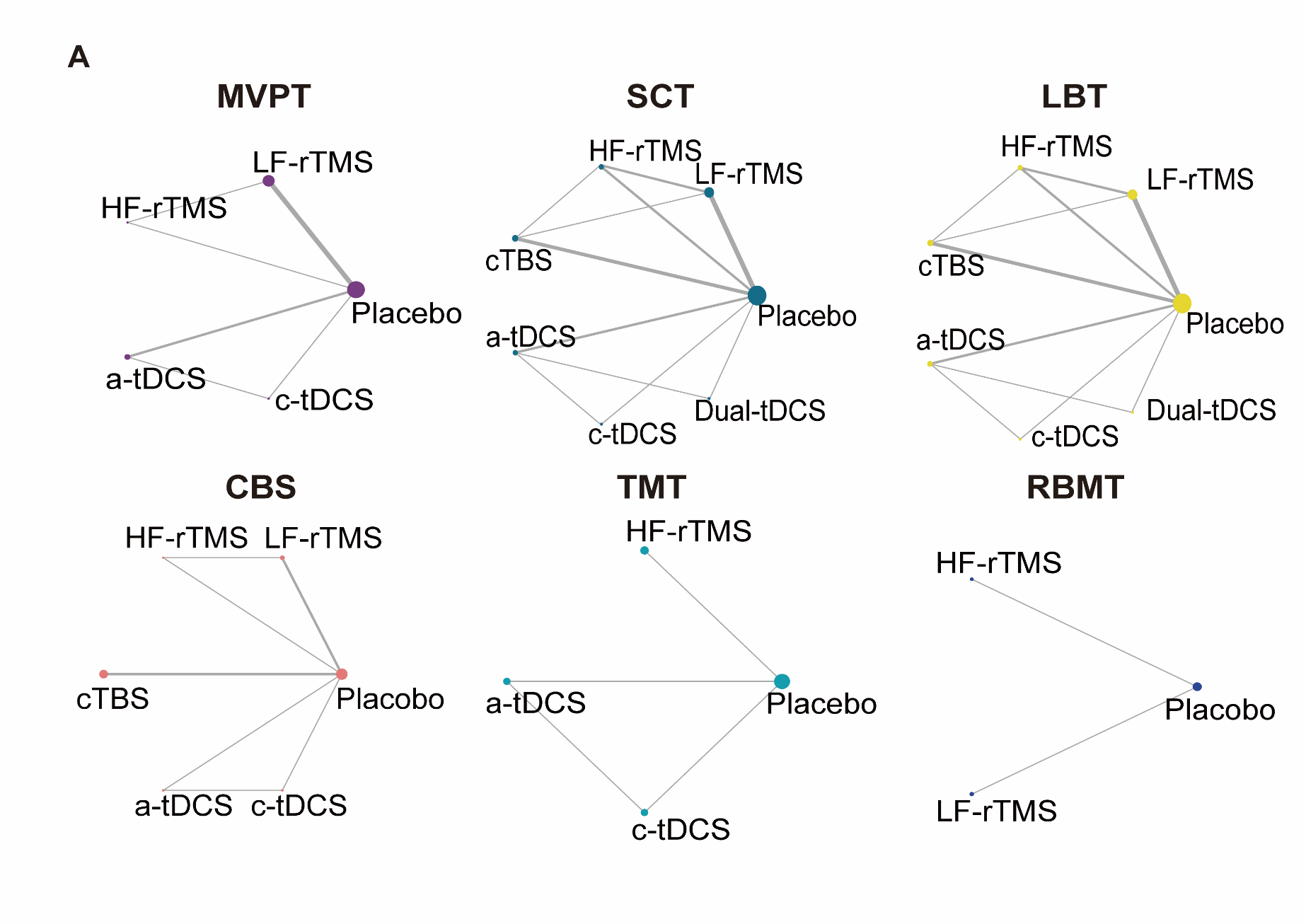
**

**
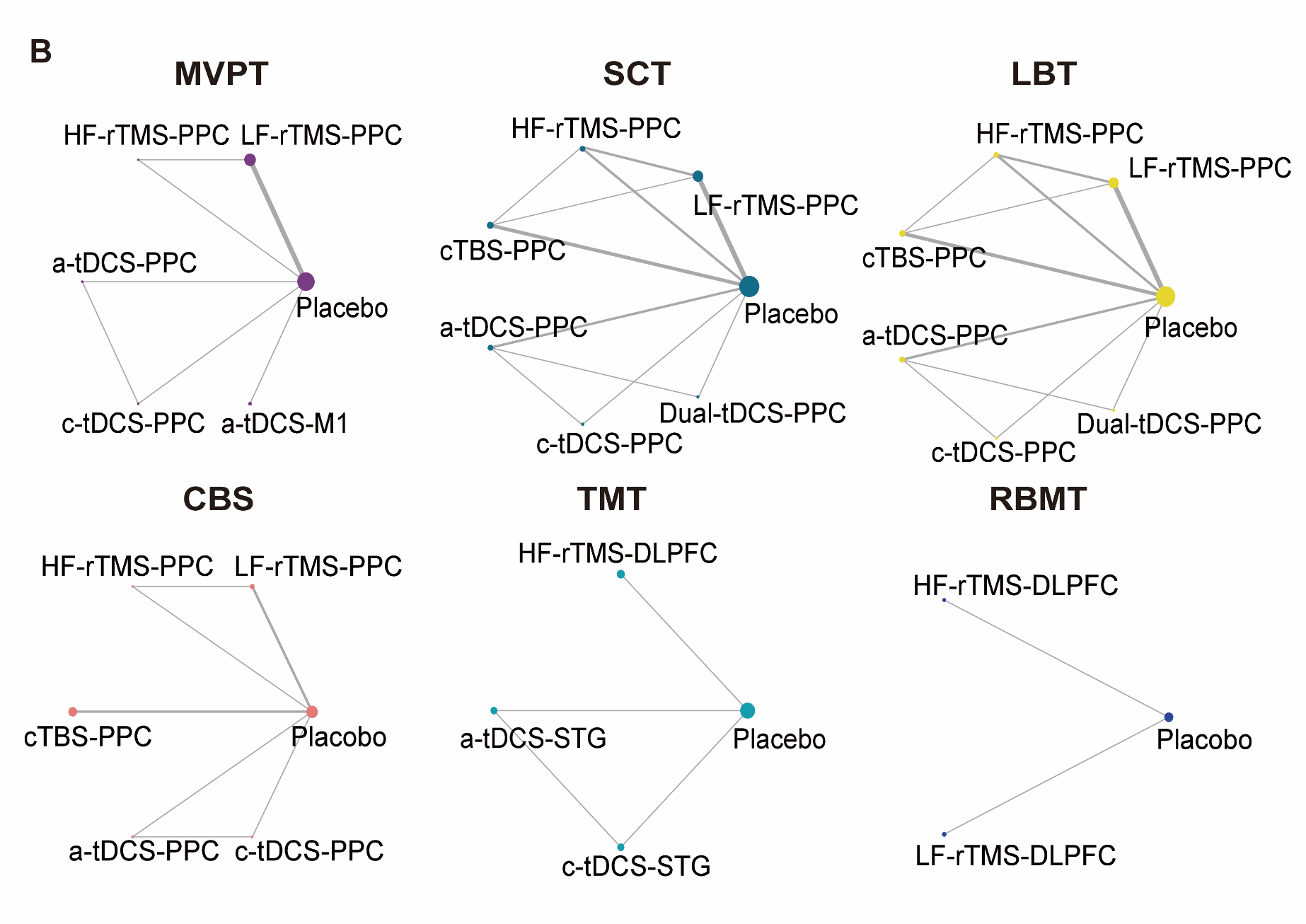
**

**Supplementary Figure 1** Network geometry of interventions across cognition function in the short-term assessment. (A) Gross network plots of NIBS modalities for cognition subdomains as defined by stimulation parameters; (B) Refined network plots of NIBS subtypes for cognition subdomains by targeted stimulation location.Each node demonstrates an intervention, and its size is weighted by the sample of patients. The edges between the nodes indicate direct comparisons of interventions, and their width is proportional to the number of trials between each pair of the comparison. HF-, high frequency; LF-, low frequency; cTBS, continuous theta burst stimulation; a-, anodal; c-, cathodal; DLPFC, dorsolateral prefrontal cortex; STG, superior temporal gyrus; PPC, posterior parietal cortex; M1, primary motor cortex; NIBS: non-invasive brain stimulation; rTMS, repetitive transcranial magnetic stimulation; tDCS, transcranial direct current stimulation.


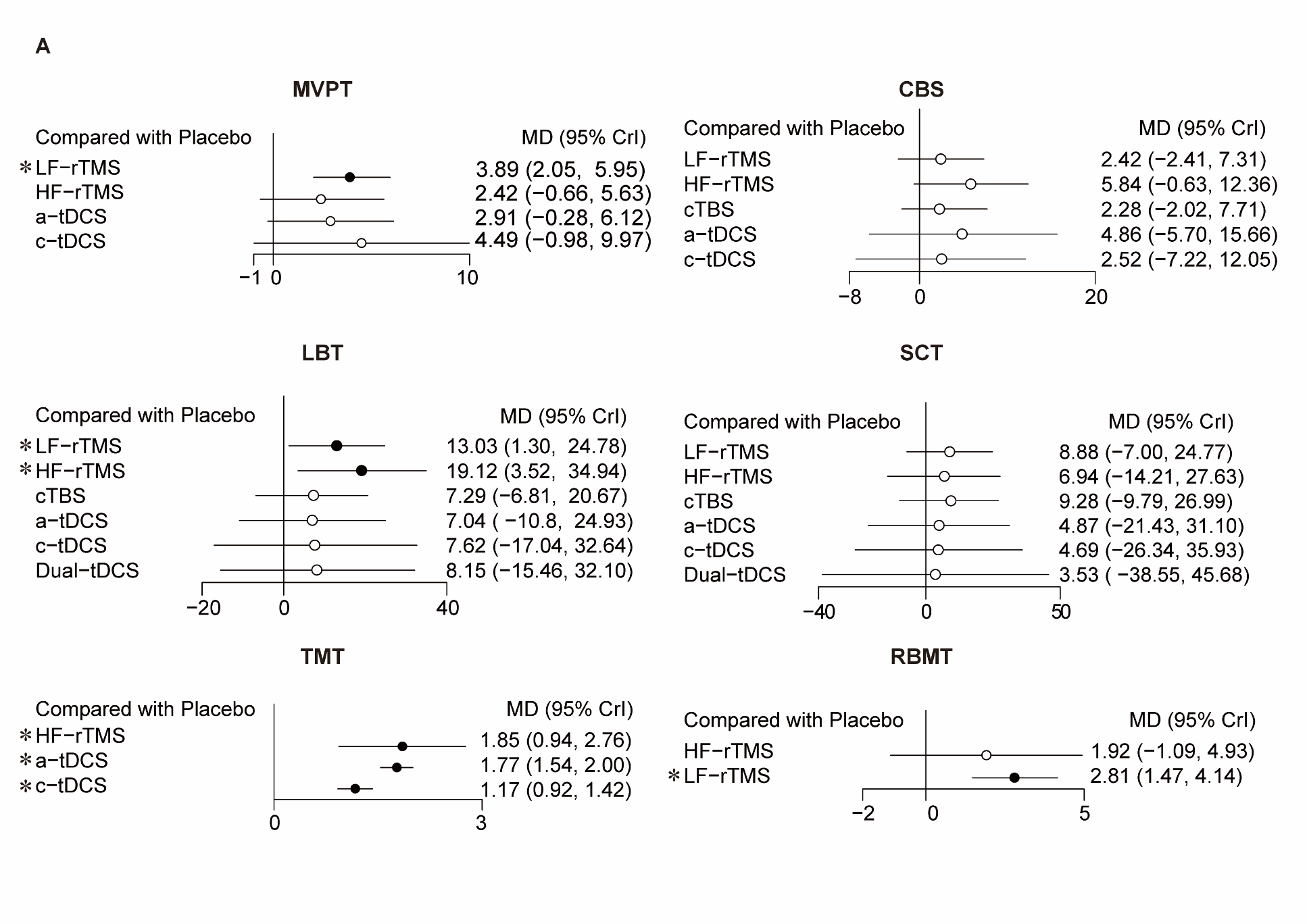


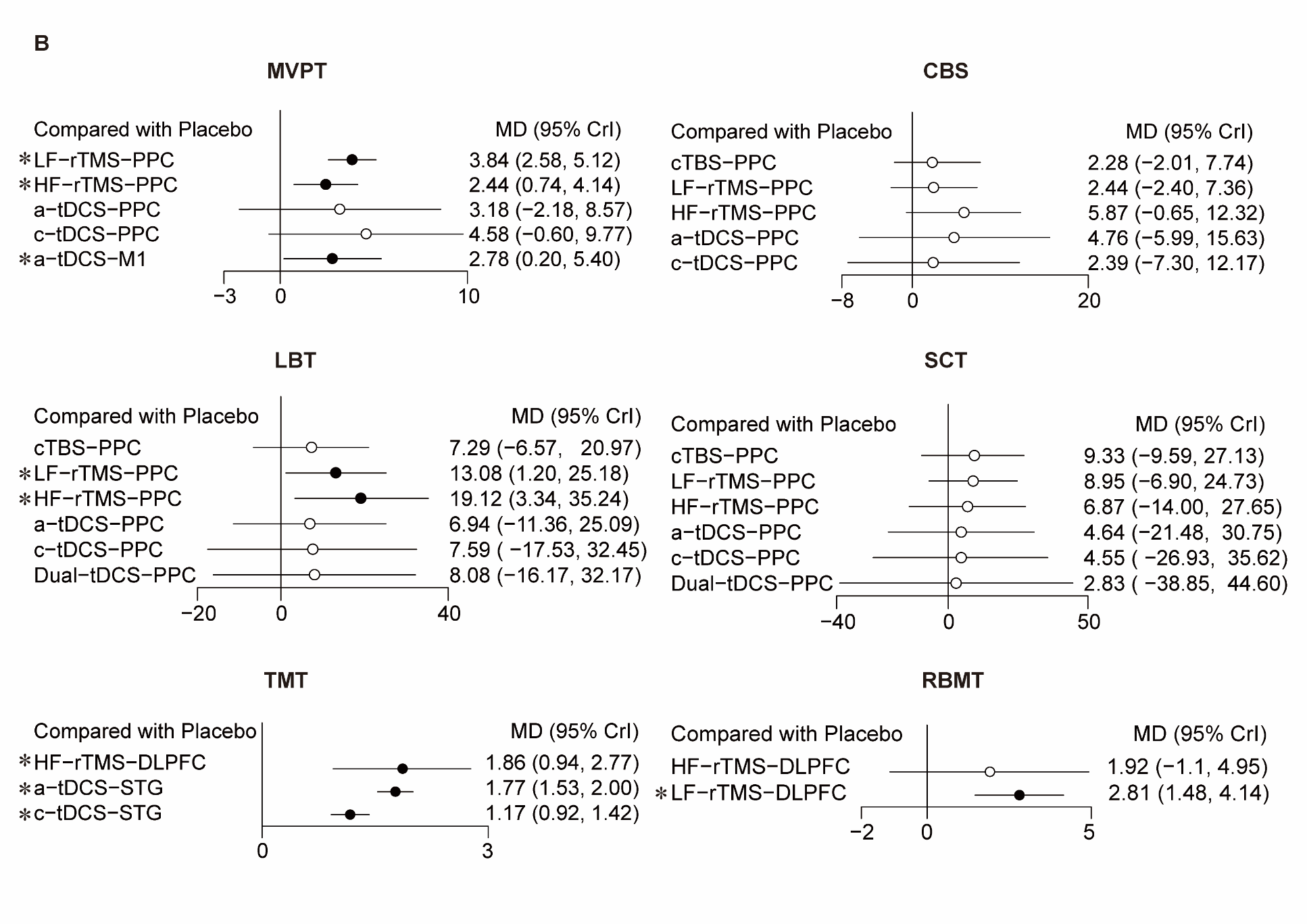


**Supplementary Figure 2** Forest plots of network meta-analyses compared with placebo across various cognition domains, pooling the effects of NIBS modalities (number of trials ≥2). (A) Forest plots of NIBS modalities for cognition subdomain as defined by stimulation parameters; (B) Forest plots of refined NIBS subtypes for cognition subdomain by targeted stimulation location. HF-, high frequency; LF-, low frequency; cTBS, continuous theta burst stimulation; a-, anodal; c-, cathodal; DLPFC, dorsolateral prefrontal cortex; STG, superior temporal gyrus; PPC, posterior parietal cortex; M1, primary motor cortex; NIBS: non-invasive brain stimulation; rTMS, repetitive transcranial magnetic stimulation; MD, mean difference; tDCS, transcranial direct current stimulation.

**Supplementary Table 2** Rankings of different interventions

| **Assessments** | **Rankings of different interventions** |
| --- | --- |
| MVPT | LF-rTMS[3.89(2.05,5.95)]＞c-tDCS＞a-tDCS＞HF-rTMS＞Placebo |
|  | LF-rTMS-PPC[3.84(2.58,5.12)]＞a-tDCS-M1[2.78(0.20,5.40)]＞HF-rTMS-PPC[2.44(0.74,4.14)]＞c-tDCS-PPC＞a-tDCS-PPC＞Placebo |
| CBS | HF-rTMS＞a-tDCS＞c-tDCS＞LF-rTMS＞cTBS＞Placebo |
|  | HF-rTMS-PPC＞a-tDCS-PPC＞LF-rTMS-DLPFC＞c-tDCS-PPC＞cTBS-PPC＞Placebo |
| LBT | HF-rTMS[19.12(3.52,34.94)]＞LF-rTMS[13.03(1.30,24.78)]＞Dual-tDCS＞c-tDCS＞cTBS＞a-tDCS＞Placebo |
|  | HF-rTMS-PPC[19.12(3.34,35.24)]＞LF-rTMS-PPC[13.08(1.20,25.18)]＞Dual-tDCS-PPC＞c-tDCS-PPC＞cTBS-PPC＞a-tDCS-PPC＞Placebo |
| SCT | cTBS＞LF-rTMS＞HF-rTMS＞a-tDCS＞c-tDCS＞Dual-tDCS＞Placebo |
|  | cTBS-PPC＞LF-rTMS-PPC＞HF-rTMS-PPC＞a-tDCS-PPC＞c-tDCS-PPC＞Dual-tDCS-PPC＞Placebo |
| TMT | HF-rTMS[1.85(0.94,2.76)]＞a-tDCS[1.77(1.54,2.00)]＞c-tDCS[1.17(0.92,1.42)]＞Placebo |
|  | HF-rTMS-DLPFC[1.86(0.94,2.77)]＞a-tDCS-STG[1.77(1.53,2.00)]＞c-tDCS-STG[1.17(0.92,1.42)]＞Placebo |
| RBMT | LF-rTMS[2.81(1.47,4.14)]＞HF-rTMS＞Placebo |
|  | LF-rTMS-DLPFC[2.81(1.47,4.14)]＞HF-rTMS-DLPFC＞Placebo |

HF-, high frequency; LF-, low frequency; cTBS, continuous theta burst stimulation; a-, anodal; c-, cathodal; DLPFC, dorsolateral prefrontal cortex; STG, superior temporal gyrus; PPC, posterior parietal cortex; M1, primary motor cortex; rTMS, repetitive transcranial magnetic stimulation; tDCS, transcranial direct current stimulation.

**
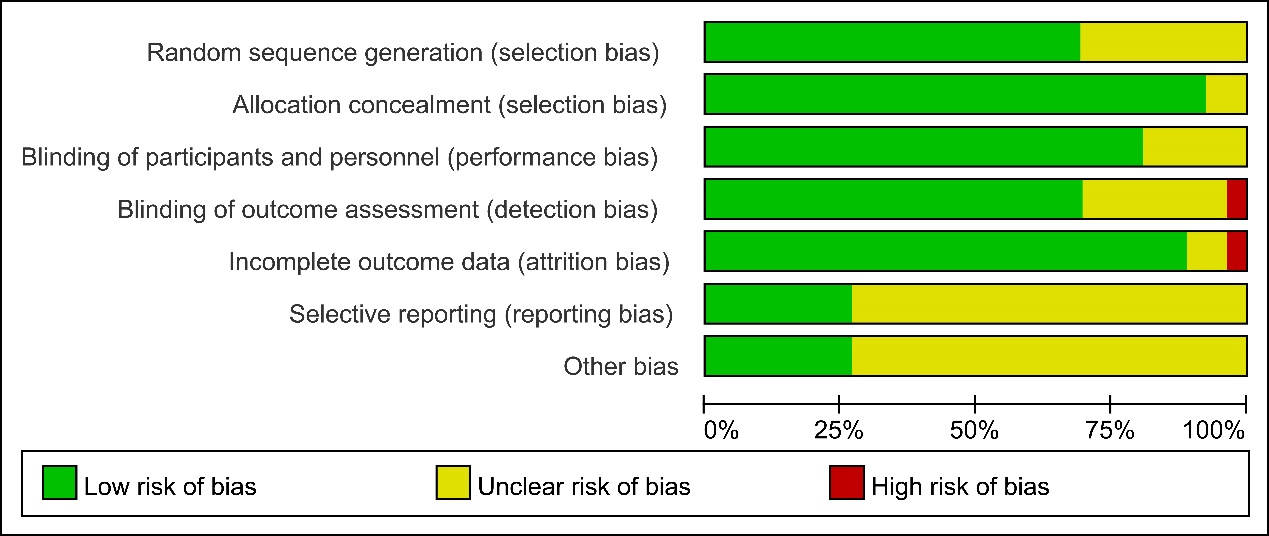
**

**Supplementary Figure 3** Risk of bias summary of included studies


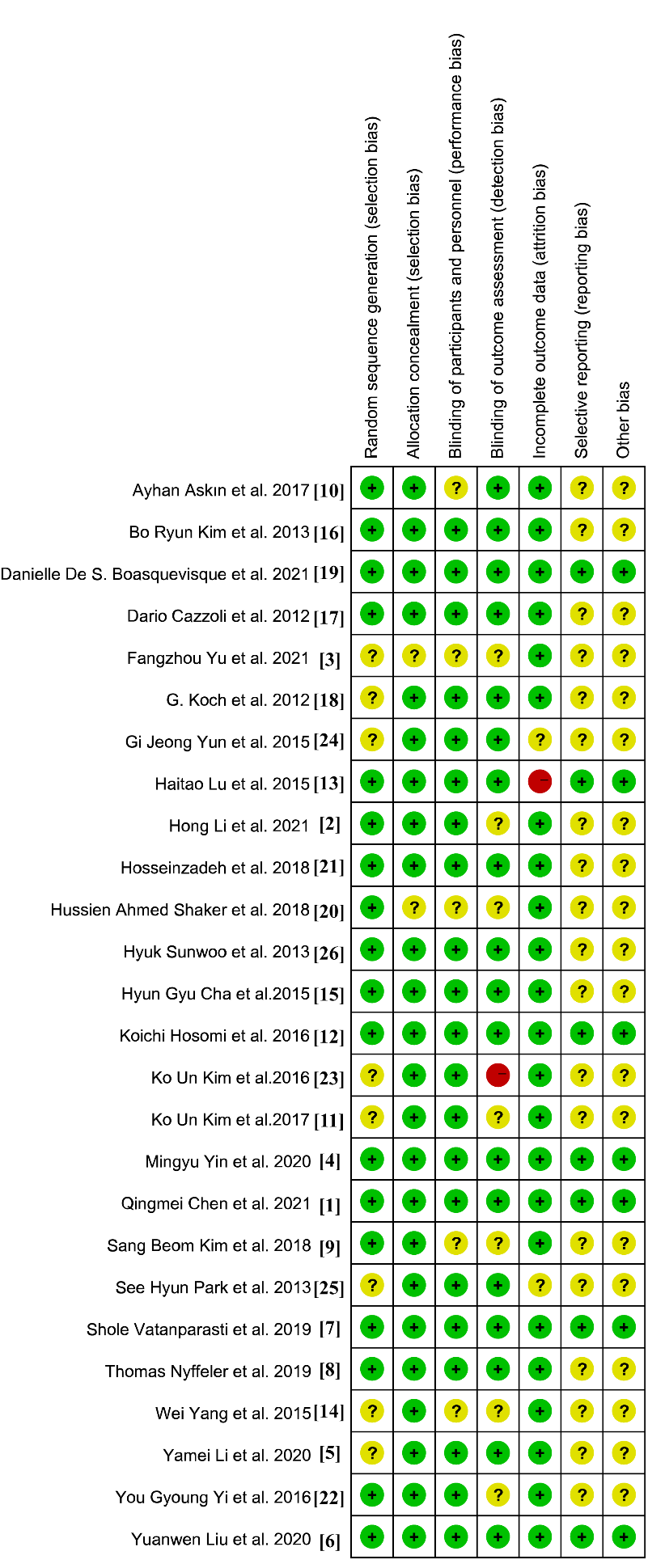


**Supplementary Figure 4** Proportion of risk levels of bias in each domain

**
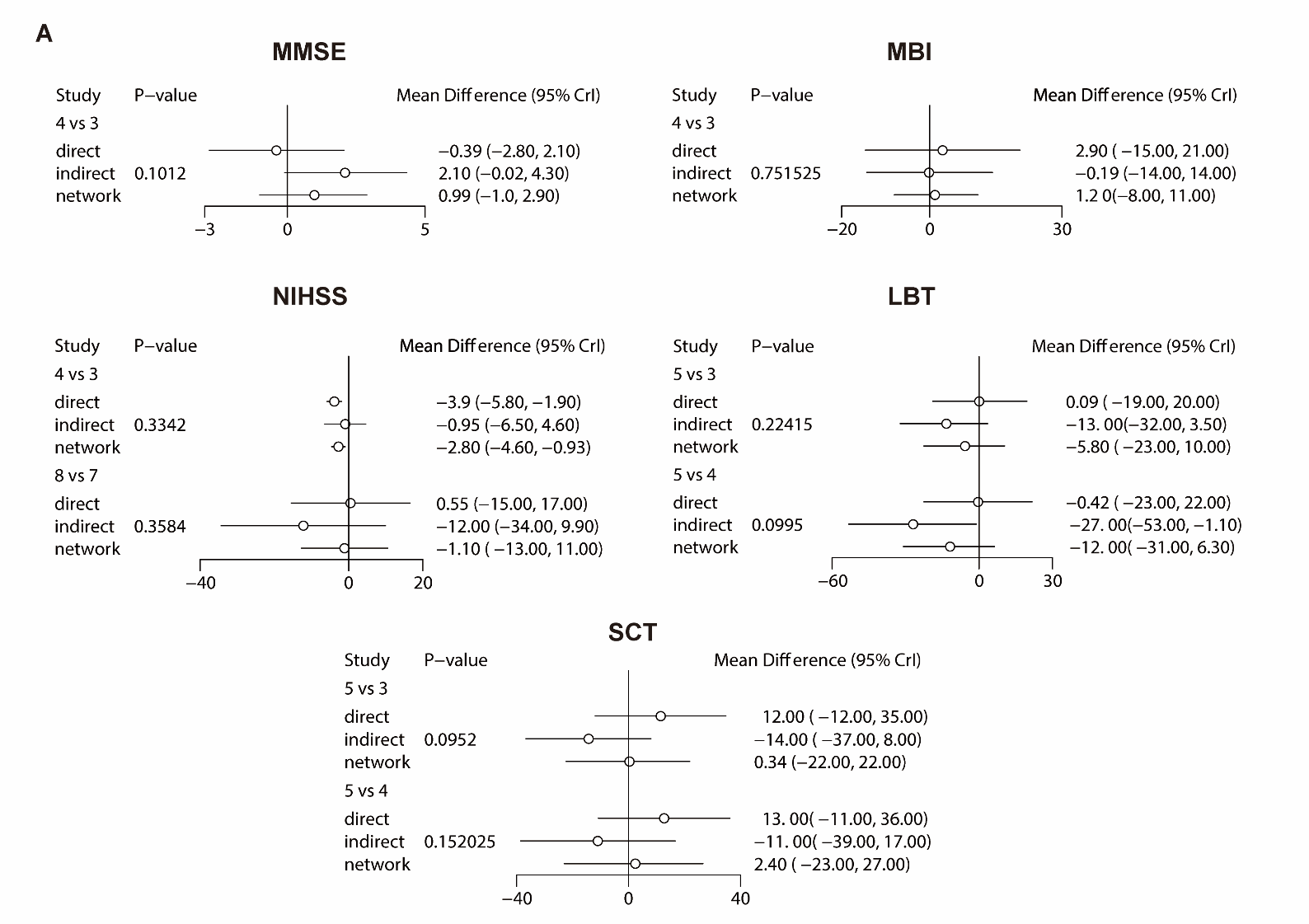
**

**
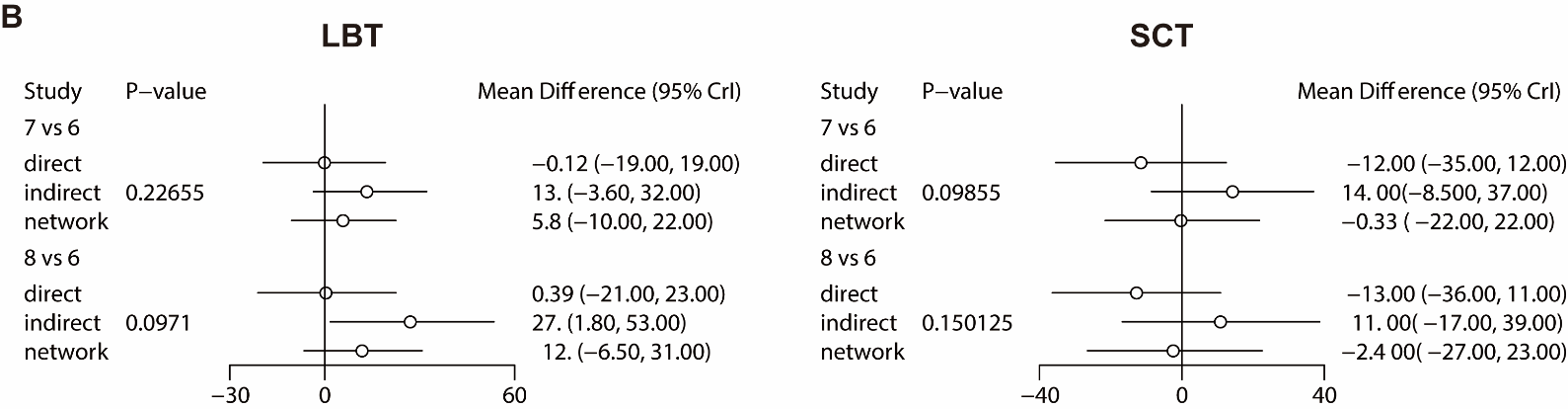
**

**Supplementary Figure 5** Evaluation of the inconsistency. (A) Plot of NIBS modalities for global severity of cognition, ADL function and cognition subdomains as defined by stimulation parameters; 3, LF-rTMS;4, HF-rTMS;5, cTBS; 7, a-tDCS;8, c-tDCS; (B) Plot of refined NIBS subtypes for global severity of cognition subdomains by targeted stimulation location; 6, cTBS-PPC;7, LF-rTMS-PPC;8, HF-rTMS-PPC; HF-, high frequency; LF-, low frequency; cTBS, continuous theta burst stimulation; a-, anodal; c-, cathodal; PPC, posterior parietal cortex; rTMS, repetitive transcranial magnetic stimulation; tDCS, transcranial direct current stimulation.

**Supplementary References**

**List of 26 Included Studies**

1. Chen, Q., et al., Effects of coupling inhibitory and facilitatory repetitive transcranial magnetic stimulation on motor recovery in patients following acute cerebral infarction. NeuroRehabilitation, 2021. 48(1): p. 83-96.

2. Li, H., et al., Repetitive Transcranial Magnetic Stimulation (rTMS) Modulates Thyroid Hormones Level and Cognition in the Recovery Stage of Stroke Patients with Cognitive Dysfunction. Medical Science Monitor : International Medical Journal of Experimental and Clinical Research, 2021. 27: p. e931914.

3. Yu, F. and R. He, The effect of fluoxetine combined with repetitive transcranial magnetic stimulation on the psychological emotions and cognitive and neurological functions of acute post-stroke depression patients. American Journal of Translational Research, 2021. 13(10): p. 11883-11889.

4. Yin, M., et al., Effects of rTMS Treatment on Cognitive Impairment and Resting-State Brain Activity in Stroke Patients: A Randomized Clinical Trial. Front Neural Circuits, 2020. 14: p. 563777.

5. Li, Y., et al., Cerebral Functional Manipulation of Repetitive Transcranial Magnetic Stimulation in Cognitive Impairment Patients After Stroke: An fMRI Study. Front Neurol, 2020. 11: p. 977.

6. Liu, Y., et al., Effects of transcranial magnetic stimulation on the performance of the activities of daily living and attention function after stroke: a randomized controlled trial. Clin Rehabil, 2020. 34(12): p. 1465-1473.

7. Vatanparasti, S., et al., The Effect of Continuous Theta-Burst Transcranial Magnetic Stimulation Combined with Prism Adaptation on the Neglect Recovery in Stroke Patients. J Stroke Cerebrovasc Dis, 2019. 28(11): p. 104296.

8. Nyffeler, T., et al., Theta burst stimulation in neglect after stroke: functional outcome and response variability origins. Brain, 2019. 142(4): p. 992-1008.

9. Kim, S.B., et al., Effect of Combined Therapy of Robot and Low-Frequency Repetitive Transcranial Magnetic Stimulation on Hemispatial Neglect in Stroke Patients. Ann Rehabil Med, 2018. 42(6): p. 788-797.

10. Askin, A., A. Tosun, and U.S. Demirdal, Effects of low-frequency repetitive transcranial magnetic stimulation on upper extremity motor recovery and functional outcomes in chronic stroke patients: A randomized controlled trial. Somatosens Mot Res, 2017. 34(2): p. 102-107.

11. Kim, K.-U., S.-H. Kim, and T.-G. An, The effects of repetitive transcranial magnetic stimulation (rTMS) on depression, visual perception, and activities of daily living in stroke patients. Journal of Physical Therapy Science, 2017. 29(6): p. 1036-1039.

12. Hosomi, K., et al., Daily Repetitive Transcranial Magnetic Stimulation for Poststroke Upper Limb Paresis in the Subacute Period. J Stroke Cerebrovasc Dis, 2016. 25(7): p. 1655-1664.

13. Lu, H., et al., Impact of repetitive transcranial magnetic stimulation on post-stroke dysmnesia and the role of BDNF Val66Met SNP. Med Sci Monit, 2015. 21: p. 761-8.

14. Yang, W., et al., Comparison of different stimulation parameters of repetitive transcranial magnetic stimulation for unilateral spatial neglect in stroke patients. J Neurol Sci, 2015. 359(1-2): p. 219-25.

15. Cha, H.G. and M.K. Kim, The effects of repetitive transcranial magnetic stimulation on unilateral neglect of acute stroke patients: A randomised controlled trial. Hong Kong Physiother J, 2015. 33(2): p. 53-58.

16. Kim, B.R., et al., Effect of high- and low-frequency repetitive transcranial magnetic stimulation on visuospatial neglect in patients with acute stroke: a double-blind, sham-controlled trial. Arch Phys Med Rehabil, 2013. 94(5): p. 803-7.

17. Cazzoli, D., et al., Theta burst stimulation reduces disability during the activities of daily living in spatial neglect. Brain, 2012. 135(11): p. 3426-3439.

18. Koch, G., et al., θ-burst stimulation of the left hemisphere accelerates recovery of hemispatial neglect. Neurology, 2012. 78(1): p. 24-30.

19. Boasquevisque, D.S., et al., Contralesional Cathodal Transcranial Direct Current Stimulation Does Not Enhance Upper Limb Function in Subacute Stroke: A Pilot Randomized Clinical Trial. Neural Plast, 2021. 2021: p. 8858394.

20. Shaker, H.A., et al., Effect of transcranial direct current stimulation on cognitive function in stroke patients. Egypt J Neurol Psychiatr Neurosurg, 2018. 54(1): p. 32.

21. Khaksarian, M., et al., Anodal transcranial direct current stimulation enhances positive changes in movement functions, visual attention and depression of patients with chronic ischemic stroke: A clinical trial. Biomedical Research and Therapy, 2018. 5(11): p. 2841-2849.

22. Yi, Y.G., et al., The Effect of Transcranial Direct Current Stimulation on Neglect Syndrome in Stroke Patients. Ann Rehabil Med, 2016. 40(2): p. 223-9.

23. Kim, K.-U., S.-H. Kim, and T.-G. An, Effect of transcranial direct current stimulation on visual perception function and performance capability of activities of daily living in stroke patients. Journal of Physical Therapy Science, 2016. 28(9): p. 2572-2575.

24. Yun, G.J., M.H. Chun, and B.R. Kim, The Effects of Transcranial Direct-Current Stimulation on Cognition in Stroke Patients. J Stroke, 2015. 17(3): p. 354-8.

25. Park, S.-H., et al., A double-blind, sham-controlled, pilot study to assess the effects of the concomitant use of transcranial direct current stimulation with the computer assisted cognitive rehabilitation to the prefrontal cortex on cognitive functions in patients with stroke. Journal of Korean Neurosurgical Society, 2013. 54(6): p. 484-488.

26. Sunwoo, H., et al., Effects of dual transcranial direct current stimulation on post-stroke unilateral visuospatial neglect. Neurosci Lett, 2013. 554: p. 94-8.
